# Multiple Stages of Metal Exsolution from Nonstoichiometric Perovskites

**DOI:** 10.1021/jacs.6c08762

**Published:** 2026-08-03

**Authors:** Andreas Rosnes, Bo Jiang, Phuong D. Nguyen, Luyang Wang, Holger von Wenckstern, Øystein Prytz, Jonathan M. Polfus

**Affiliations:** † Department of Physics, Centre for Materials Science and Nanotechnology, 6305 University of Oslo, P.O. Box 1048 Blindern, NO NO-0316 Oslo, Norway; ‡ Department of Chemistry, Centre for Materials Science and Nanotechnology, University of Oslo, P.O. Box 1033 Blindern, NO-0315 Oslo, Norway

## Abstract

Exsolution enables controlled solid-state precipitation of metal nanoparticles from metal oxides, resulting in materials that exhibit excellent catalytic properties for electrochemical energy conversion. The diverse characteristics of particles exsolved at different temperatures indicate complex mechanisms that remain unresolved. Through a series of in situ electron microscopy, synchrotron X-ray diffraction, and thermogravimetry experiments on La_0.2_Ca_0.7_Ti_0.9_Sc_0.05_Cu_0.05_O_3−δ_ across nanoscale wedges to μm-sized powders, multiple stages of exsolution are deconvoluted with increasing thermal activation: (1) minor exsolution of anchored surface nanoparticles, (2) exsolution of endoparticles within the bulk, and (3) diffusion and coalescing in the bulk and at the surfaces. Reconstruction of the perovskite surface by formation of nanoscopic steps and terraces proceeds concurrently with endoparticle growth. Moreover, distinct exsolution characteristics are observed in the near-surface region and deeper within the bulk, resulting in spherically and orthorhombically shaped endoparticles, respectively.

## Graphical abstract

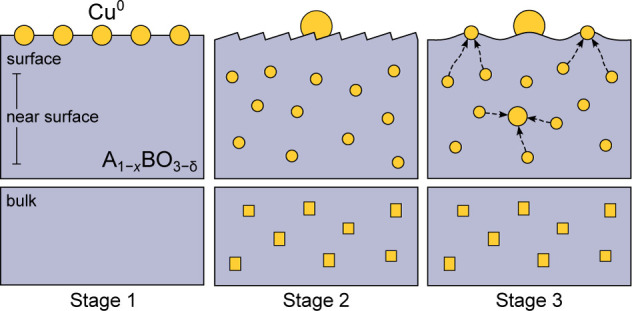

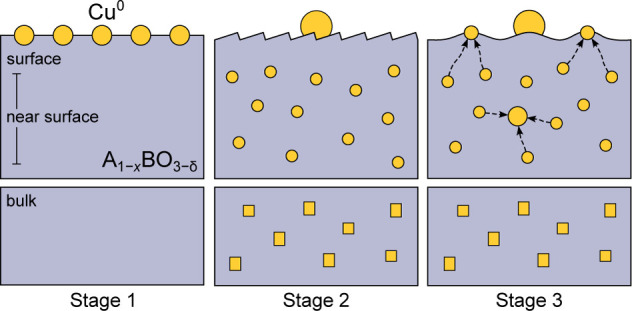

## Introduction

Exsolution enables controlled solid-state precipitation of finely dispersed metal nanoparticles from metal oxide substrates,^1–3^ triggered by a reductive treatment at elevated temperatures.^4–6^ The strong interaction between the nanoparticles and host metal oxide inhibits agglomeration, yielding catalysts with high activity and durability suitable for catalytic and electrochemical applications.^7–10^

Central to the catalytic properties of the nanoparticles are their characteristics in terms of size, shape, and interface to the host metal oxide.^11,12^ The anchoring of exsolved nanoparticles is primarily attributed to socket formation associated with the reverse Schottky reaction, describing annihilation of host unit cells by removal of point defects amounting to one formula unit.^13^ Strongly anchored nanoparticles of B-site constituents can therefore be achieved by controlling the A-site deficiency in perovskite oxides (A_1–*x*_BO_3−δ_).^3^ For instance, 15–20 nm sized surface nanoparticles can be stabilized at temperatures as high as 900 °C in nonstoichiometric La_0.2_Sr_0.7_Ti_0.9_Ni_0.1_O_3−δ_ thin films.^14–16^ Further, the exsolution yield can be significantly enhanced by addition of trivalent B-site acceptors to charge balance the perovskite after the reaction while also leading to increased nucleation density of nanometric particles in aluminum- and gallium-substituted La_0.2_Ca_0.7_Ti_0.95_Cu_0.05_O_3−δ_.^17^ Formation and growth of surface nanoparticles can proceed by lattice diffusion and reduction of the exsolving species.^18^ Alternatively, metal particles exsolved within the metal oxide matrix, denoted endoparticles, can emerge and coalesce at the surfaces, as evidenced in La_0.4_Sr_0.4_Ti_0.97_Ni_0.03_O_3−δ_ between 600 and 900 °C^19^. Bimodal particle populations—consisting of surface nanoparticles and endoparticles—are reported for A-site-deficient perovskite titanates.^2,20^ The strength of the anchoring to the metal oxide support differentiates the coalescing mechanism, which may proceed by either particle migration if the metal–support interaction is weak or by Ostwald ripening if the interaction is strong.^21^ The size distribution, surface density, and socketing depth of the particles can be further modified by the surface termination,^14^ lattice strain,^15^ or point defect concentrations.^22^

Conversely, stoichiometric perovskites lack A-site vacancies, which disfavors the reverse Schottky reaction, thereby promoting exsolution of nanoparticles mainly at surfaces^23–26^ or grain boundaries.^16^ Consequently, the particles exhibit limited anchoring and coalesce at notably lower temperatures than in nonstoichiometric perovskites.^27^ Surface nanoparticles with diameters of 15–20 nm can be stabilized by donor doping at nonpolar (100) facets of stoichiometric SrTi_0.9_Nb_0.05_Ni_0.05_O_3−δ_ thin films at 400–700 °C.^28^ The mechanisms of exsolution from stoichiometric perovskites remain elusive in terms of accompanying changes in structure and stoichiometry, e.g., segregation of A-rich minority phases,^29^ partial decomposition,^30^ or formation of B-site vacancies and extended defects such as Ruddlesden–Popper faults.^31–33^ Moreover, exsolution from SrTi_0.65_Fe_0.35_O_3−δ_ thin films at 800 °C leads to iron depletion from a ∼2 nm surface layer accompanied by titanium enrichment and formation of polycrystalline regions.^25^ In contrast to the nonstoichiometric systems, the exsolution yield appears determined not by bulk point defects but rather by the chemistry of the metal oxide surface as exsolution is limited to the near-surface region, leaving the majority of the transition metal within the bulk.^24,25^

The diverse characteristics of exsolved nanoparticles in stoichiometric and nonstoichiometric perovskites—including bimodal particle populations—indicate distinct exsolution mechanisms that are initiated at different temperatures. Here, the exsolution process is resolved into three stages by a series of in situ experiments using scanning transmission electron microscopy (STEM), thermogravimetry, and synchrotron X-ray diffraction (XRD). Accordingly, the stages are identified as (1) minor exsolution of surface nanoparticles, (2) exsolution of endoparticles, and (3) diffusion of metal nanoparticles or species to the surface and coalescing, initiating at approximately >230 °C, >450 °C, and >750 °C, respectively. Moreover, distinct endoparticle characteristics are observed in the near-surface regions in nanoscale wedges compared to the bulk of μm-sized powders. The reducibility of copper and fast exsolution kinetics of La_0.2_Ca_0.7_Ti_0.9_Sc_0.05_Cu_0.05_O_3−δ_ and La_0.2_Ca_0.7_Ti_0.9_Al_0.05_Cu_0.05_O_3−δ_ are utilized to differentiate the exsolution stages,^34–36^ exploiting a relatively minor lattice mismatch between the perovskite and the exsolved metal, excess A-site vacancies,^3^ and inherent oxygen vacancies due to the scandium and aluminum acceptors.^17^

## Results

### Sequential Stages of Exsolution

Exsolution involves reduction of the perovskite by release of oxygen gas, allowing indirect insight into the reaction by monitoring the mass loss using thermogravimetry. Three distinct oxygen release stages occur upon heating to 1000 °C under reducing conditions (2 °C/min, 4% H_2_). The stages initiate at ∼230 °C, ∼450 °C, and ∼750 °C, respectively (Figure 1a), determined by the onsets of increased rate of mass change. The mass loss curve is corrected for buoyancy, and mass loss due to partial reduction of Ti^4+^ to Ti^3+^ occurs above 900 °C in the copper-free reference (Figure S1). The residual mass loss processes are thereby assigned to exsolution.

**Figure 1:**
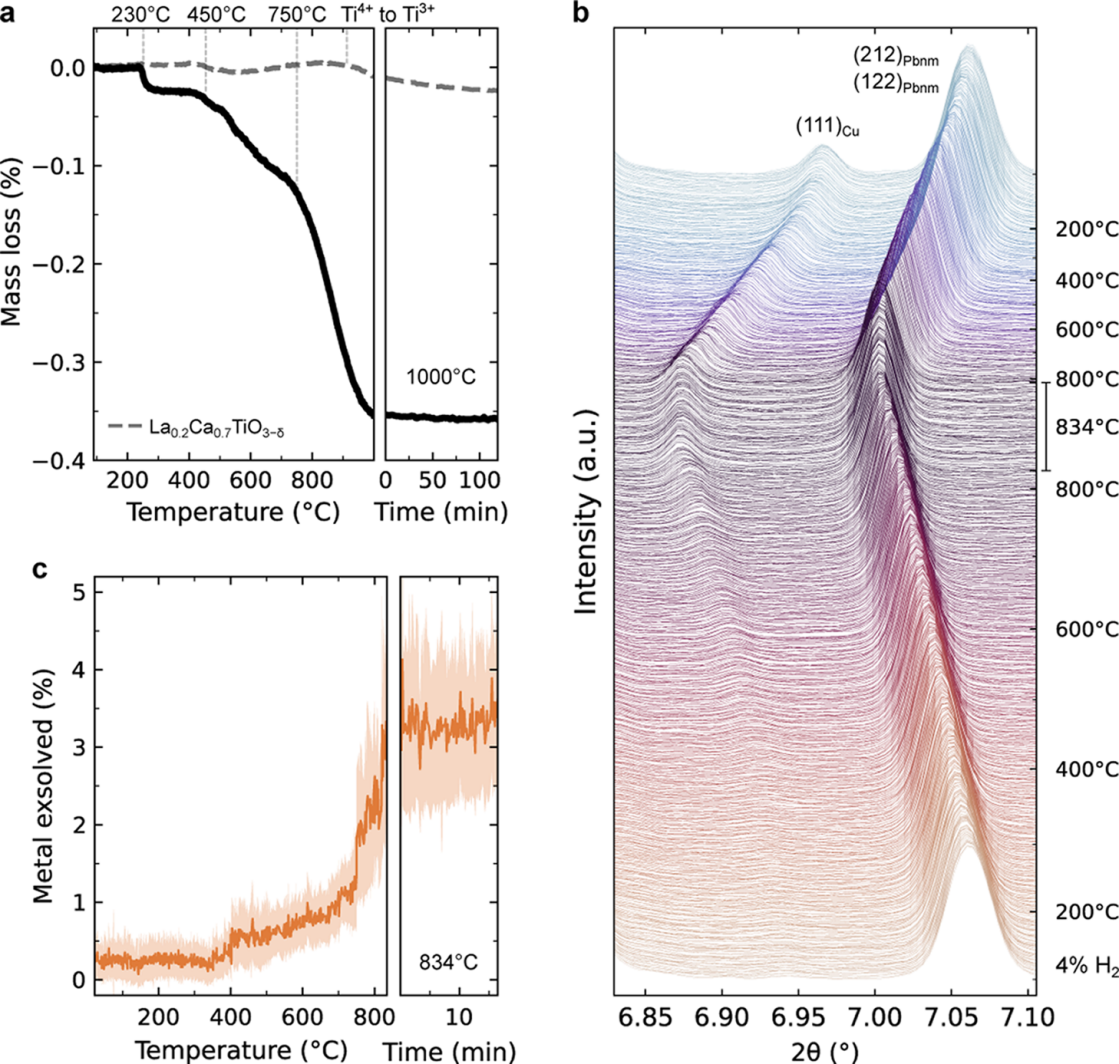
Temperature-resolved stages of copper exsolution in La_0.2_Ca_0.7_Ti_0.9_Sc_0.05_Cu_0.05_O_3−δ_ from macroscopic techniques: In situ characterization of copper exsolution in La_0.2_Ca_0.7_Ti_0.9_Sc_0.05_Cu_0.05_O_3−δ_ thermogravimetry up to 1000 °C and synchrotron XRD up to 834 °C, respectively, in 4% H_2_. (a) Thermogravimetric analysis shows three mass-loss processes associated with exsolution after correction using a La_0.2_Ca_0.7_TiO_3−δ_ reference. Dashed vertical lines mark estimated onset temperatures. (b) Synchrotron XRD upon heating and subsequent cooling shows minor copper exsolution initializing at ∼350 °C. (c) Estimated amount of exsolved metal by the resolvable phases from Rietveld refinement of the diffractograms.

Exsolved copper is directly characterized by synchrotron XRD under a similar reductive treatment up to 834 °C (30 °C/min, 4% H_2_), as shown in the magnified part of the diffractograms encapsulating the Cu (111) and perovskite (212) and (122) reflections (Figure 1b). The onset of exsolution occurs at ∼350 °C according to Rietveld refinement (Figure 1c). Notably, both thermogravimetry and XRD show that most of the copper exsolves at temperatures above 700 °C. The perovskite structure is maintained throughout the experiment with negligible changes in symmetry and lattice parameters in line with the relatively minor exsolution yield (Figures S3 and S4).

The macroscopic insights were used to guide the in situ STEM experiments, enabling atomic-scale observations of the formation and evolution of the particle population. Drop-casted powder samples were studied at temperatures up to 1000 °C in ultrahigh vacuum (10^–8^ mbar) and quenched to room temperature (200 °C/s) for high-resolution imaging. Three stages of exsolution are identified: (1) exsolution of surface nanoparticles, (2) nucleation and growth of endoparticles, and (3) diffusion and coalescing in the bulk and at the surfaces, initiating at ∼400 °C, ∼600 °C, and ∼1000 °C, respectively, highlighted with representative STEM annular dark field (ADF) images from a <110>-oriented wedge (Figure 2a). Atomically resolved images further show a nanoparticle at the perovskite surface (Figure 2b), endoparticles together with a reconstructed surface (Figure 2c), and ultimately a smoothed surface (Figure 2d). The findings were reproduced on <100>- and <111>-oriented wedges and on a <100>-oriented wedge of La_0.2_Ca_0.7_Ti_0.9_Al_0.05_Cu_0.05_O_3−δ_, as well as in regions not previously exposed to the electron beam irradiation (Figures S5–S12).^37^

**Figure 2:**
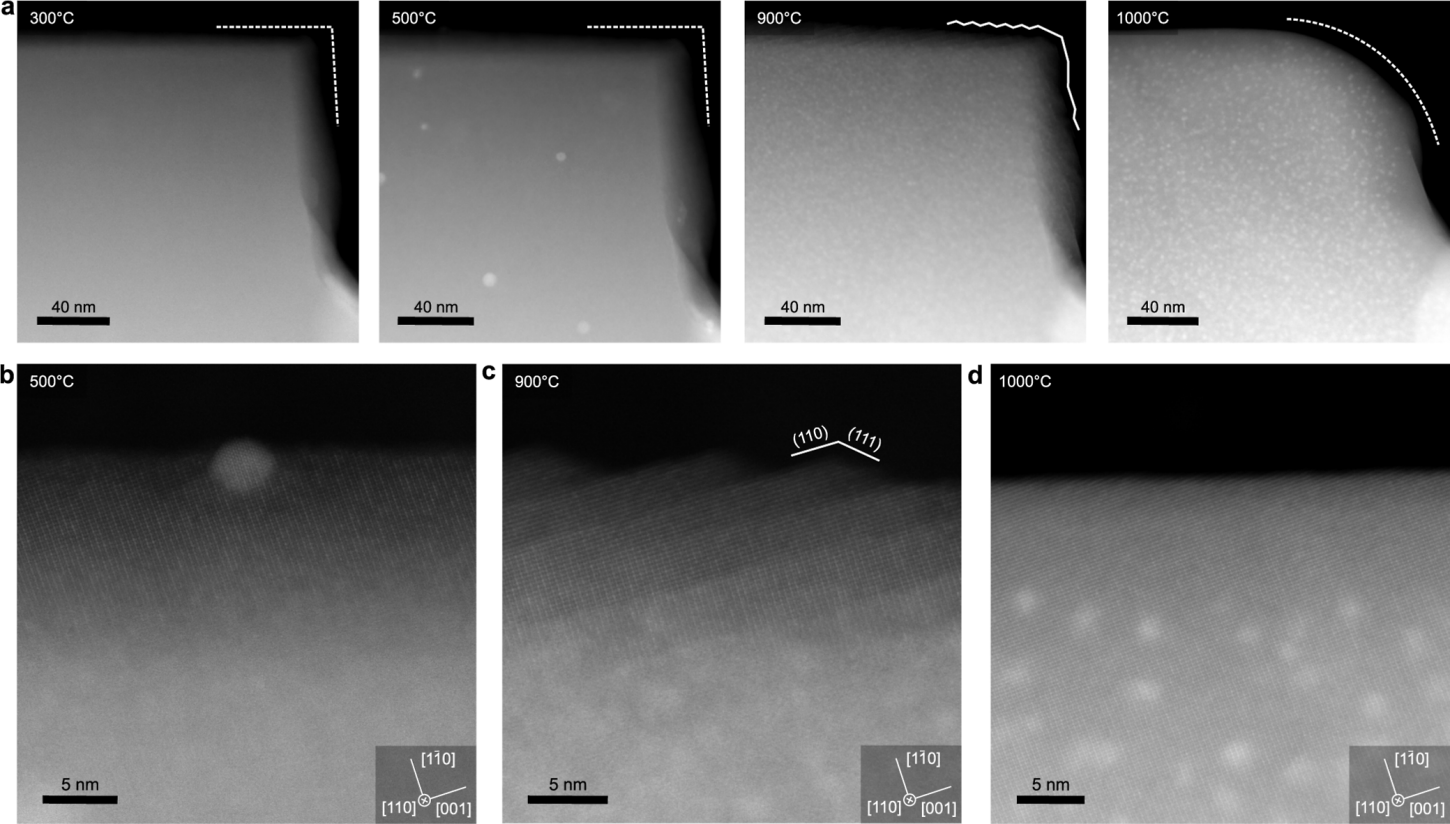
Temperature-resolved stages of exsolution observed by in situ STEM ADF imaging of La_0.2_Ca_0.7_Ti_0.9_Sc_0.05_Cu_0.05_O_3−δ_ under ultrahigh vacuum (10^–8^ mbar) up to 1000 °C. (a) Nanoscale images of a wedge show multiple stages of exsolution upon heating to 1000 °C: (1) surface nanoparticles (500 °C), (2) endoparticle formation and surface reconstruction (900 °C), and (3) diffusion and coalescing of nanoparticles concurrent with surface smoothening (1000 °C). (b–d) Atomic-scale STEM ADF images of (b) a surface nanoparticle (500 °C), (c) surface reconstruction by step and terrace formation (900 °C), and (d) the nanoparticle population following endoparticle lattice diffusion (1000 °C).

Consistency with macroscopic techniques was evaluated by performing the thermogravimetry experiment for perovskite particles with increasing diameters up to 200 μm, revealing that the mass loss in the initial stage scales approximately with the specific surface area (Figure 3). The onset temperature decreases with decreasing particle size, consistent with enhanced exsolution from fracture surfaces compared to the native surfaces of the sintered material.^3^ The onset temperatures from in situ STEM are somewhat higher than in the XRD and TG experiments, which can be ascribed to the milder reductive treatment and different heating rates.

**Figure 3:**
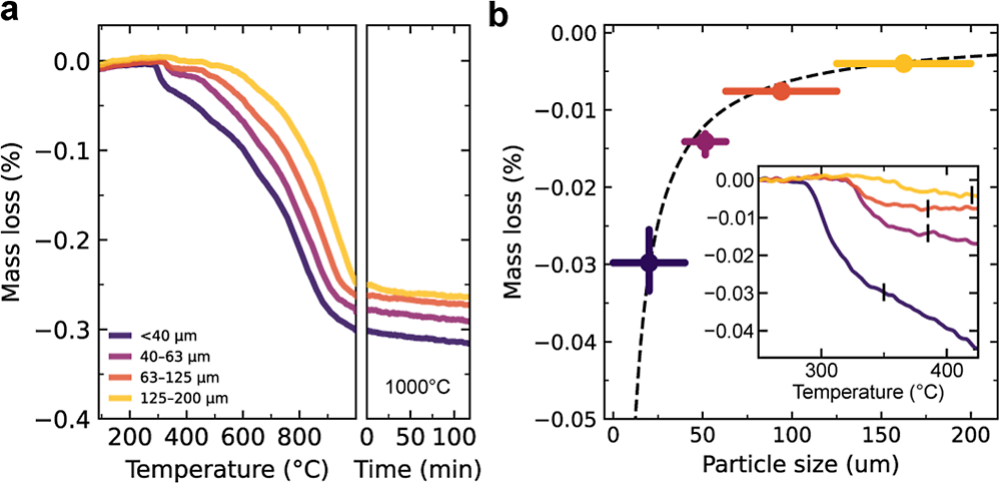
Thermogravimetric analysis of La_0.2_Ca_0.7_Ti_0.9_Sc_0.05_Cu_0.05_O_3−δ_ powders with increasing particle size up to 200 μm in diameter. (a) Mass loss curves after correction using a La_0.2_Ca_0.7_TiO_3−δ_ reference. The differences in final mass loss correspond well with the differences in the initial stage. (b) Mass loss in the initial stage (60 °C after the onset) scales approximately with the specific surface area, i.e., proportional to the reciprocal diameter. Horizontal lines represent the particle size fraction, and vertical lines represent the mass loss at 40–80 °C after the onset.

### Initial Exsolution of Surface Nanoparticles

A sample wedge close to the low-order zone axis <110> with a thickness of about 100 nm was used for the in situ STEM experiment. After a 1 min anneal at 500 °C, nanoparticles exsolve and stabilize with diameters of 5.5 ± 1.5 nm with no detectable agglomeration after 20 min, indicating sufficient anchoring to counteract particle diffusion and coalescing (Figures 4a and S6). Moreover, images of particles at the wedge edge corroborate that exsolution occurs at the surface (Figure 4b).

**Figure 4:**
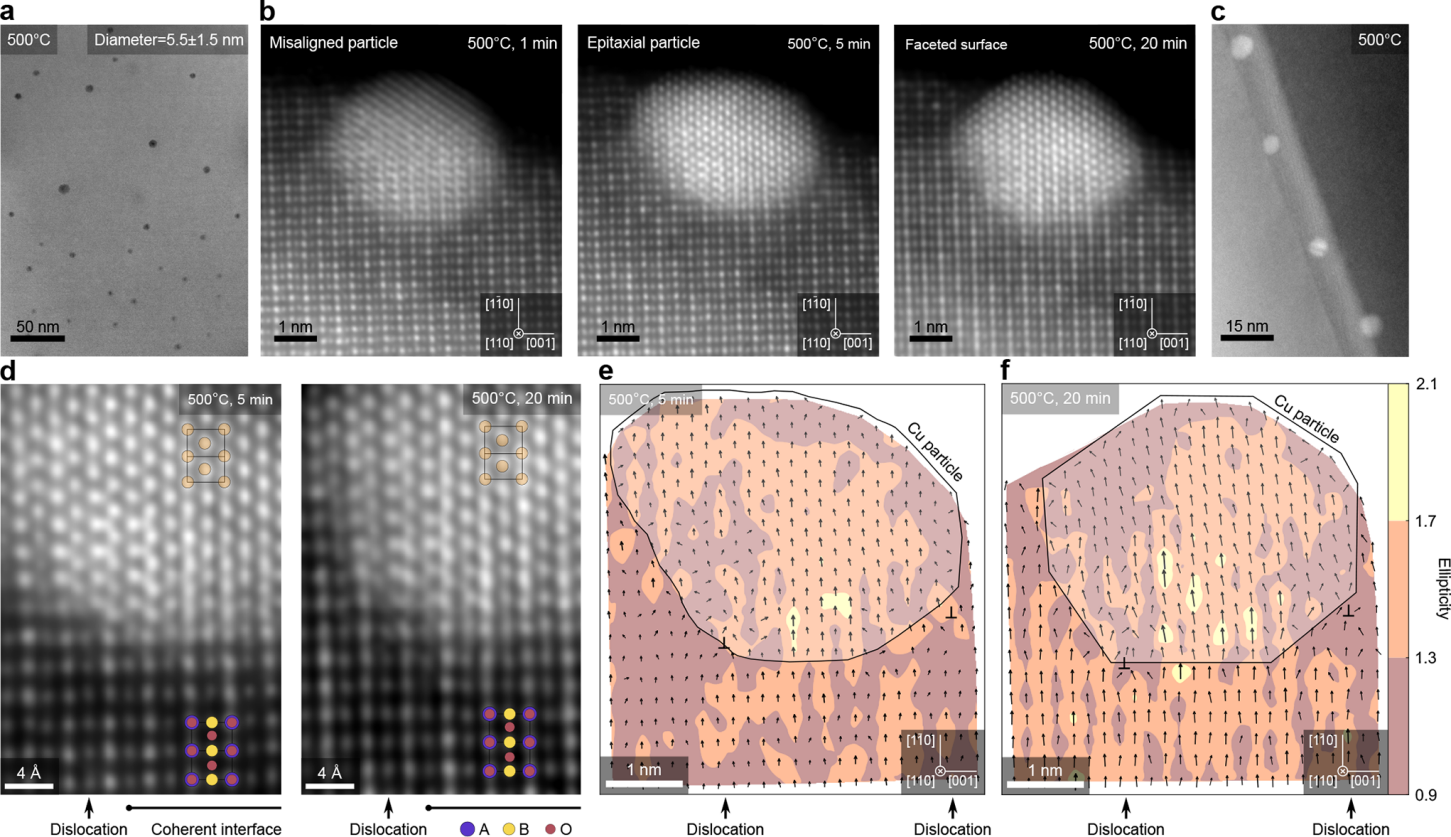
Surface exsolution in La_0.2_Ca_0.7_Ti_0.9_Sc_0.05_Cu_0.05_O_3−δ_ under ultrahigh vacuum (10^–8^ mbar) after annealing at 500 °C. (a) Overview STEM annular bright-field image of the particle population of the <110>-oriented wedge with diameters of 5.5 ± 1.5 nm obtained from 30 particles. The image is flattened by a linear background to visualize nanoparticles across the interior of the wedge with varying thickness. (b) Side-view STEM ADF image of surface nanoparticles acquired after 1, 5, and 20 min, showing a misaligned, epitaxial, and faceted particle, respectively. (c) STEM ADF image of nanoparticles at a grain boundary. (d) Magnified image of the metal–perovskite interface region after 5 and 20 min shows a semicoherent interface with lattice mismatch and a misfit dislocation. Annotations visualize fcc and pseudocubic perovskite unit cells oriented on the <110> zone axis. (e,f) Combined vector and contour plots of the elliptical distortion as obtained by 2D Gaussian fitting of the atomic columns after 5 and 20 min. The shaded area indicates the copper particle.

Atomically resolved images of an exsolved surface particle after 1, 5, and 20 min provide further insight into the evolution of the structural relationship to the support (Figure 4b). The nanoparticle initially crystallizes incoherently with the perovskite matrix, as seen by the misaligned atomic columns. Epitaxy develops after 5 min by alignment between the face-centered cubic (fcc) copper structure and the AO_3_ network of the perovskite with analogous symmetry, visually accessible along the [110] direction. Finally, the metal forms well-defined facets after 20 min.

The (pseudo)­cubic lattice parameter of the perovskite is significantly larger than that of copper, 3.82 Å and 3.61 Å, respectively, from Rietveld refinement of the X-ray diffractograms at room temperature. The difference in lattice parameter initially gives rise to a semicoherent interface characterized by significant misfit strain and dislocations (Figure 4d). The misfit strain in the interface region is relieved in the vicinity of the dislocation, visualized by overlaid vector and contour plots of refined atomic column positions wherein elliptical distortion indicates the local level of strain (Figure 4e).^38^ After 20 min, the stabilized particle shows increased lattice deformation around the dislocation, mainly in the copper metal with altered spacing of the (002) planes (Figure 4f). Nanoparticles are additionally observed at grain boundaries (Figure 4c), which provide a higher degree of freedom for structural relaxation.

### Exsolution of Endoparticles and Surface Reconstruction

Overview STEM ADF images of the wedge and its corner reveal that the exsolved surface nanoparticles disappear from view after 1 min at 700 °C, presumably coalescing to larger nanoparticles in nearby regions (Figure 5a). Subsequently, a new contrast mechanism appears in the interior regions of the wedge and becomes increasingly prominent up to 900 °C (Figure 5a,b). Under the STEM ADF imaging conditions, contrast is enhanced by point defects and local lattice distortion due to diffuse scattering and dechanneling of the electron wave.^39,40^ The contrast extends over a few unit cells in the atomically resolved image, implying the presence of point defect clusters, likely to be subnanometric copper nuclei (Figure 5b). Delocalization of the contrast combined with overlap of the nanoparticles within the wedge projection prevents accurate quantification of their size and position within the wedge. A minor defocus is introduced to enhance the contrast around the nanoparticles, which appears to extend not more than 1 nm in the images at lower magnification (Figure 5b). In combination with the onset of copper reduction and metallic phase formation at similar temperatures from thermogravimetry and X-ray diffraction, respectively, these observations are consistent with the exsolution of copper endoparticles during the second stage.

**Figure 5:**
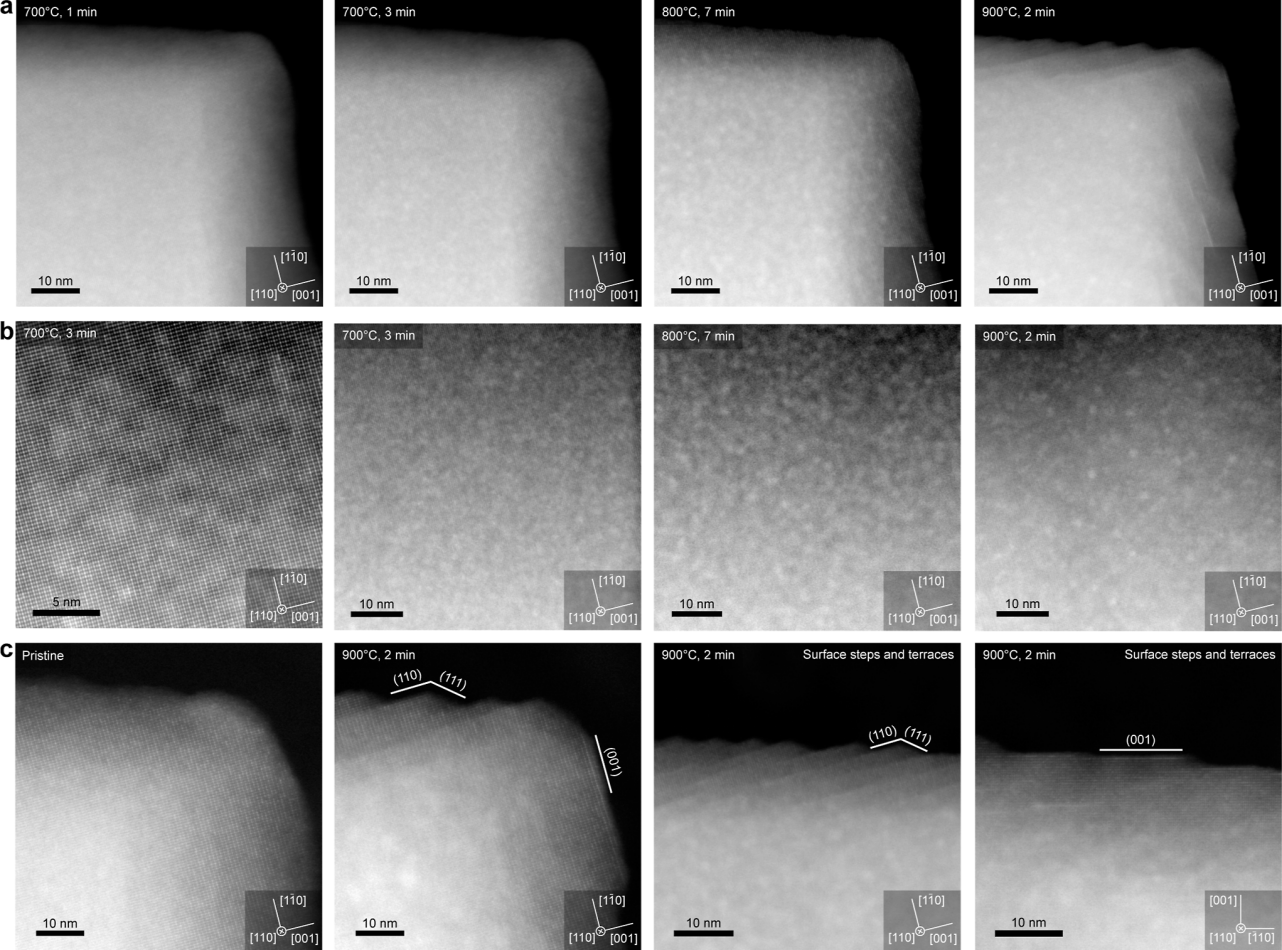
STEM images of endoparticle exsolution in La_0.2_Ca_0.7_Ti_0.9_Sc_0.05_Cu_0.05_O_3−δ_ under ultrahigh vacuum (10^–8^ mbar) after annealing at 700 to 900 °C. (a) STEM ADF image of the wedge corner shows the absence of the initially exsolved surface nanoparticles and the evolution of a new contrast in the bulk between 700 and 900 °C together with surface reconstruction at 900 °C. (b) Magnified STEM ADF images of the bulk lattice show the contrast evolving in a fully retained host metal oxide. (c) The wedge surface undergoes reconstruction by the formation of steps and terraces with (100), (110), and (111) terminations.

The perovskite surface undergoes significant reconstruction, forming steps and terraces after 2 min at 900 °C (Figure 5c). The low-order zone axis provides a direct view of the reconstruction, resulting in (110)/(111)- and (001)-terminated facets at the horizontal and vertical sides of the wedge, respectively. The (001) terraces become comparatively longer and show enrichment of lanthanum, reflected by the brighter A-site columns at the AO-terminated surface.

### Endoparticle Diffusion and Surface Smoothening

After 5 min at 1000 °C, the wedge and nanoparticle population have undergone further changes (Figure 6). The particle population exhibits an average diameter of 2.0 ± 0.3 nm, indicating particle growth compared to the previous stage. Further, newly formed nanoparticles are observed at the surface, clearly evident at the wedge edge where the surface is directly exposed (Figure 6b). These observations are consistent with activation of endoparticle diffusion through the perovskite lattice toward the surfaces,^19^ while growth of the surface particles may also proceed via diffusion of single species.^21^

**Figure 6:**
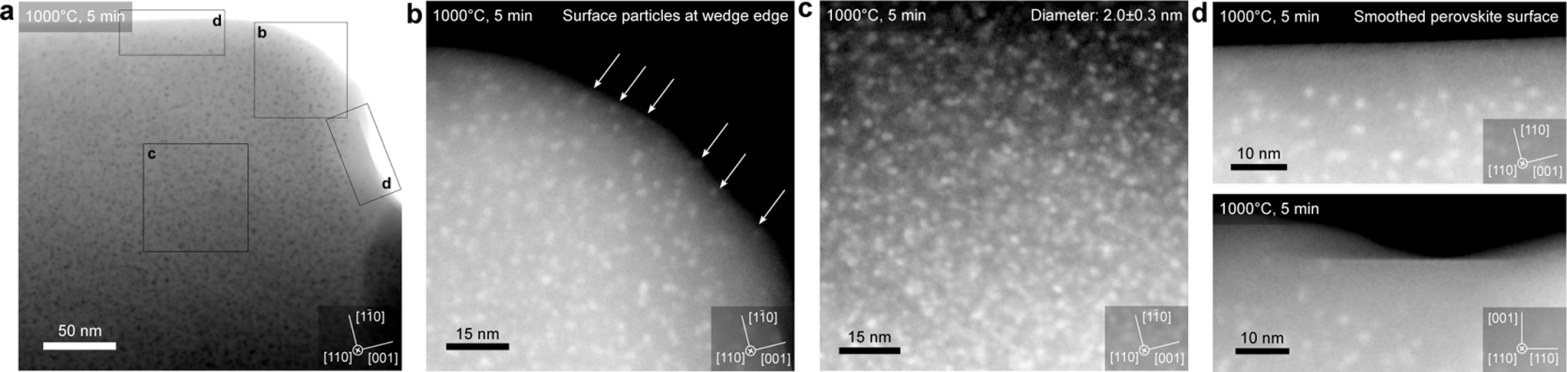
STEM images of exsolution and surface smoothening in La_0.2_Ca_0.7_Ti_0.9_Sc_0.05_Cu_0.05_O_3−δ_ under ultrahigh vacuum (10^–8^ mbar) after 1000 °C. (a) Overview STEM annular bright field image of the <110>-oriented wedge showing the nanoparticle population and a smoothed surface. (b) Nanoscale STEM ADF image in off-wedge geometry show surface nanoparticles at the wedge edge. (c) Magnified STEM ADF image of the particle population, representing an average diameter of 2.0 ± 0.3 nm as measured from 50 particles. (d) Local variation in the surface nanoparticles, correlated to the previously observed step and terrace formation. The regions in (b–d) are marked in (a).

The surface steps and terraces of the perovskite are concurrently smoothed,^18^ implying increased diffusion of the host cations at these temperatures. Local variations in the density of nanoparticles are observed in the thin regions previously dominated by steps and terraces (Figure 6d). Diffusion and coalescing of the surface nanoparticles are observed above 1000 °C in La_0.2_Ca_0.7_Ti_0.9_Al_0.05_Cu_0.05_O_3−δ_ (Figure S12), further supporting that endoparticles ultimately end up at the surface at this stage.

### Endoparticle Shape Determined by Proximity to the Perovskite Surface

The in situ STEM experiments are performed on thin wedges with a relatively high surface-to-bulk ratio, meaning that the surface area is large relative to the amount of exsolved copper endoparticles in the near-surface region. The characteristics of the endoparticles within thicker regions were studied in powders with diameters of 20–40 μm that were reduced at 750 °C in 5% H_2_ for 24 h and subsequently crushed to reveal wedges of their interior.^17^ ADF and annular bright field (ABF) images reveal orthorhombically shaped nanoparticles, elongated along the principal crystallographic directions (Figure 7a,b). The nanoparticle sizes are estimated from their cross-section to an average spherical diameter of 3.6 ± 0.6 nm. The plane of focus varies across the particle population due to their depth distribution, confirming that the particles reside within the bulk. Moreover, strain fields along the [001] direction are visualized with perpendicular extinction lines positioned on the nanoparticles in the ABF image at the <120> zone axis (Figure 7c). Crucially, the orthorhombically shaped nanoparticles are remarkably different from the spherical particles in the near-surface regions studied in the in situ experiments (Figure 3).

**Figure 7:**
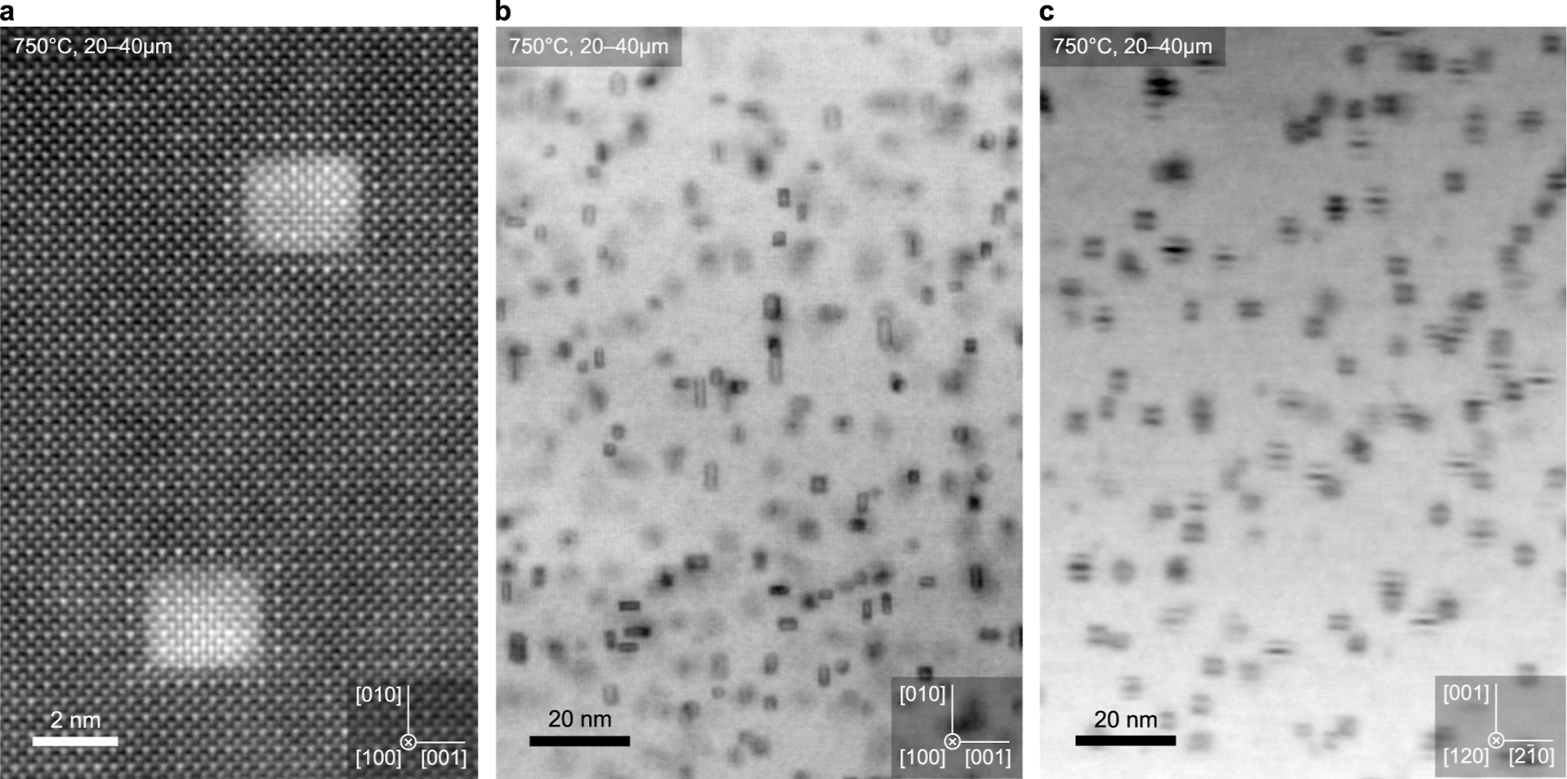
Orthorhombically shaped endoparticles in the subsurface regions of La_0.2_Ca_0.7_Ti_0.9_Sc_0.05_Cu_0.05_O_3−δ_ particles of 20–40 μm exsolved at 750 °C under 4% H_2_ for 24 h. (a) Atomically resolved STEM ADF image showing strained copper particles. (b,c) Low-magnification STEM ABF images of the endoparticle population show orthorhombically shaped particles elongated along the principal crystallographic directions with extinction lines perpendicular to [001] on the <120> zone axis.

## Discussion

The distinct stages of exsolution are anticipated to manifest differently across material systems. Nonstoichiometric perovskites that contain all point defects required for the reverse Schottky reaction enable all stages, including endoparticle formation and diffusion, as in La_0.4_Sr_0.4_Ti_0.97_Ni_0.03_O_3−δ_.^19^ The exsolution yield is therefore determined by the availability of point defects according to the exsolution reaction, as long as strain does not dominate.^17^ Conversely, stoichiometric perovskites appear to predominantly allow the initial stage of surface exsolution in the absence of structural changes that accommodate the B-site deficiency, in line with most reductive treatments being below 700 °C in SrTi_0.65_Fe_0.35_O_3−δ_ ^23,24^ and SrTi_0.95_Ni_0.05_O_3−δ_.^26–28^ The minor exsolution yield of this stage implies that the majority of the transition metal remains within the perovskite lattice. Notably, the sequential stages are consistent with the isothermal evolution of exsolved copper from in situ synchrotron XRD, showing initial exsolution of unstrained particles that develop tensile strain within the first 5 min at 800 °C.^17^ These observations are consistent with activation of all three stages of exsolution, resulting in a bimodal particle population comprising predominantly unstrained surface nanoparticles and strained nanoparticles originating from the bulk. The onset temperature of each exsolution stage may be strongly influenced by the lattice mismatch between the perovskite and the exsolving metal.^17^

During the initial exsolution stage, anchoring develops via epitaxy and relaxation of strain from lattice mismatch and misfit dislocations at the metal–perovskite interface (500 °C, Figure 4). The restricted cation mobility in the perovskite at these temperatures indicates that socketing is limited to reorganization of the surface in the proximate region of the nucleation site. In contrast, anchoring of the surface nanoparticles originating from endoparticles develops in conjunction with larger-scale rearrangement of the surface, as seen with smoothening of steps and terraces above 1000 °C (Figures 5 and 6). Anchoring due to microstructural rearrangement in the same temperature range has been observed by elevation of the perovskite surface directly around nickel particles in La_0.43_Ca_0.37_Ti_0.94_Ni_0.06_O_3−δ_ at 900 °C.^20,34^ Remarkably, the stabilized particles are smaller above 1000 °C (2.0 ± 0.3 nm) than those formed at the initial stage below 600 °C (5.5 ± 1.5 nm), implying stronger anchoring despite the larger-scale rearrangement of host cations.

The observed surface reconstruction may be linked to the exsolution of the endoparticles (Figure 4). Strain imposed due to the lattice mismatch between the endoparticles and the host induces stress, which may be relieved by spontaneous surface reconstruction.^41,42^ Morphological changes at the surface can also be linked to the overall exsolution reaction, including the reverse Schottky reaction, which involves reduction of one transition metal atom per removed perovskite formula unit.^17^ Accordingly, four perovskite unit cells are annihilated per exsolved fcc copper unit cell. Notably, a corresponding excess void volume is absent in atomically resolved images of the endoparticles (Figure 7a),^17^ implying that the excess Schottky defects annihilate at the surfaces or remain within the bulk lattice. The surface reconstruction may thereby be facilitated by the annihilation of the unit cells. The observed surface reconstruction may also be largely independent of exsolution, as the sample preparation exposes fracture surfaces that can reconstruct to lower the surface energy.

Observations of spherically and orthorhombically shaped nanoparticles in the near-surface regions of the nanoscale wedges (Figure 6) and the thicker regions of the μm-scale powders (Figure 7), respectively, imply distinct exsolution characteristics, despite the identical stoichiometry and lattice mismatch. The maximization of the (100), (010), and (001) copper–perovskite interfaces of the orthorhombically shaped nanoparticles indicates growth governed by strain (Figure 7). The near-surface regions examined during in situ STEM exhibit short diffusion lengths for the removal of excess Schottky defects, in addition to the possibility of locally alleviating strain through surface reconstruction. Both mechanisms are suppressed in the thicker regions of the μm-scale powders, leading to buildup of extensive strain upon exsolution and growth of highly strained orthorhombic endoparticles. Strain, moreover, limits the exsolution yield compared to equivalent systems with lower lattice mismatch where spherical endoparticles also appear within thicker regions of the bulk.^17^

## Conclusions

The study allows three main conclusions to be drawn regarding exsolution from nonstoichiometric perovskites, exemplified by La_0.2_Ca_0.7_Ti_0.9_Sc_0.05_Cu_0.05_O_3−δ_. First, exsolution proceeds via three principal stages activated at increasing temperatures: (1) exsolution of surface nanoparticles, (2) nucleation and growth of endoparticles, and (3) diffusion and coalescing in the bulk and at the surfaces. Second, surface reconstruction occurs together with endoparticle growth and may alleviate strain in the near-surface region. Third, the exsolution characteristics depend on strain, leading to spherically shaped endoparticles in the near-surface region and highly strained orthorhombically shaped particles deeper within the bulk.

## Methods

### Materials

Solid-state reaction synthesis was used to prepare La_0.2_Ca_0.7_Ti_0.9_Acc_0.05_Cu_0.05_O_3−δ_ with acceptors Acc = {Al, Sc} from stoichiometric amounts of La_2_O_3_ (99.9%), CaCO_3_ (>99.9%), TiO_2_ (99.8%), CuO (99.99%), Al_2_O_3_ (>99.9%), and Sc_2_O_3_ (99.9%). The lanthanum sesquioxide powder was dried for 10 h at 1000 °C, quenched, and placed in a desiccator before weighing. The powders were ball-milled in 5 min intervals for 3 h at 350 rpm in an agate jar (Retsch PM100) before consecutive calcinations at 650 and 950 °C for 3 h, ball-milled similarly for 5 h, cold-pressed to 1-in. disks, and sintered at 1500 °C for 10 h in ambient air. The sintered disks were crushed and sieved for further characterization.

### Synchrotron X-ray Diffraction and Absorption Spectroscopy

In situ studies were performed at beamline BM31 at the European Synchrotron Radiation Facility (ESRF).^46^ Glass capillaries of 0.7 mm diameter and wall thickness of 0.01 mm were filled with powders of diameter in the range 125 to 200 μm to ensure sufficient gas flow during the experiment. The powders were heated at 50 °C increments to 834 °C in 4% H_2_ in a He atmosphere with a gas flow of 10 mL/min with a ramp rate of 30 °C/min and 100 °C/min during heating and cooling, respectively. The powders were continually exposed to the synchrotron X-rays for XRD (λ = 0.25402 Å) measurement during the ramping stage. Refinements were performed in Topas v6 Academic^43^ with further results presented in Figures S3 and S4. X-ray diffraction maps were collected on a PILATUS X CdTe 2 M detector.^44^ XAS measurements were conducted at isothermal dwells at the 50 °C increment in fluorescence mode during the in situ experiment.

### Thermogravimetric Analysis

In situ mass loss during exsolution was measured using a Netzsch STA449F3 Jupiter thermogravimetric analyzer with powder samples of about one-half gram and particle size fractions <40 μm, 40–63 μm, 63–125 μm, and 125–200 μm. The sample was heated to 300 °C with a ramp rate of 10 °C/min in N_2_ followed by an isothermal dwell to remove any contamination. The mass loss was then stabilized at room temperature before 5% H_2_ in an Ar atmosphere with a flow rate of 40 mL/min and a protective gas of 10 mL/min N_2_, which combine to a 4% H_2_ mixture. The measured mass loss, temperature profile, and gas profiles are presented in Figures S1 and S2. A blank run under identical conditions with an empty crucible and powder of La_0.2_Ca_0.7_TiO_3−δ_ was used to correct for buoyancy and evaluate minor reduction of the host oxide in the absence of the exsolution reaction, respectively.

### In Situ Nanoscale Imaging and Spectroscopy

A FEI Titan G2 60–300 TEM instrument with a DCOR Cs probe corrector operated at 200 kV was used to perform nanoscale imaging. The in situ experiments were performed using a Protochips Fusion MEMS-based heating stage within the TEM column with an ultrahigh vacuum of 10^–8^ mbar. As-synthesized powders were crushed in an agate mortar with high-purity isopropanol (99.9%) and drop-casted onto the Protochip heating microchips with 18 nm carbon-coated films with 2 μm sized windows. Prior to the sample dispersion, the chip was heat treated overnight at 150 °C in air to remove surface contamination and improve sample dispersion on the support film. The microchips were then baked overnight at 150 °C in air after sample drop-casting as well as a 20 min preanneal at 200 °C inside the TEM column to remove contaminations prior to beam exposure.

An overview of the in situ experiments, additional results, and reproduced experiments on La_0.2_Ca_0.7_Ti_0.9_Sc_0.05_Cu_0.05_O_3−δ_ and La_0.2_Ca_0.7_Ti_0.9_Al_0.05_Cu_0.05_O_3−δ_ are provided in Figures S5–S12. Electron beam irradiation may impact the observed exsolution phenomena, providing an additional driving force for the reaction.^37^ The influence of the electron beam was evaluated in the initial experiments and mitigated by performing imaging mainly at room temperature.

ADF STEM images were obtained using a convergence angle of 24.8 mrad with an inner collection angle of 27.9 mrad and a probe current of 0.1 nA from an XFEG source controlled with a Wien-filter monochromator. The deep convolutional neural network developed by Lobato et al.^45^ was used to remove experimental noise of selected atom-resolved images. Data handling was performed in ecosystems of the Hyperspy package,^48^ where Atomap was used to refine the atomic column positions and ellipticities using 2D Gaussian.^3^ STEM energy-dispersive X-ray spectroscopy (EDS) was obtained using a Super-X EDS detector system with a nominal collection solid angle of 0.7 srad (Figures S6 and S11). Spectral images were denoised and reconstructed using principal component analysis.^47^

## Supplementary Material



## Data Availability

Data from TG, XRD, and STEM analysis are available at the Zenodo repository at https://doi.org/10.5281/zenodo.20714376.

## Notes

Thermogravimetric analysis (TGA), structure characterization and refinement (XRD), temperature profiles of in situ microscopy experiments (STEM), exsolution at the initial stage (STEM), endoparticle formation and surface reconstruction (STEM), and diffusion and coalescing in the bulk and at the surface (STEM) (PDF)
