# Supplementary material for: Multiple Stages of Metal Exsolution from Nonstoichiometric Perovskites
Source: J Am Chem Soc. 2026 Aug 3;148(31):33486–95. doi: 10.1021/jacs.6c08762 (PMC13474946; doi:10.1021/jacs.6c08762)
Supplement: Supplementary file 1 [file ja6c08762_si_001.pdf]

Supplementary Information

Multiple stages of metal exsolution from non-stoichiometric perovskites

Andreas Rosnes[1,*], Bo Jiang[2], Phuong D. Nguyen[1], Luyang Wang[2], Holger von Wenckstern[1], Øystein Prytz[1], Jonathan M. Polfus[2,*]

[1] Department of Physics, Centre for Materials Science and Nanotechnology, University of Oslo, PO Box 1048 Blindern, NO NO-0316 Oslo, Norway

[2] Department of Chemistry, Centre for Materials Science and Nanotechnology, University of Oslo, PO Box 1033 Blindern, NO-0315 Oslo, Norway

* Corresponding authors E-mail: andreas.rosnes@smn.uio.no, jonathan.polfus@kjemi.uio.no

Contents

1. Thermogravimetric analysis (TGA) 2

2. Structure characterization and refinement (XRD) 4

3. Temperature profiles of in situ microscopy experiments (STEM) 6

4. Exsolution at the initial stage (STEM) 7

5. Endoparticle formation and surface reconstruction (STEM) 10

6. Diffusion and coalescing in the bulk and at the surface (STEM) 11

1. Thermogravimetric analysis (TGA)

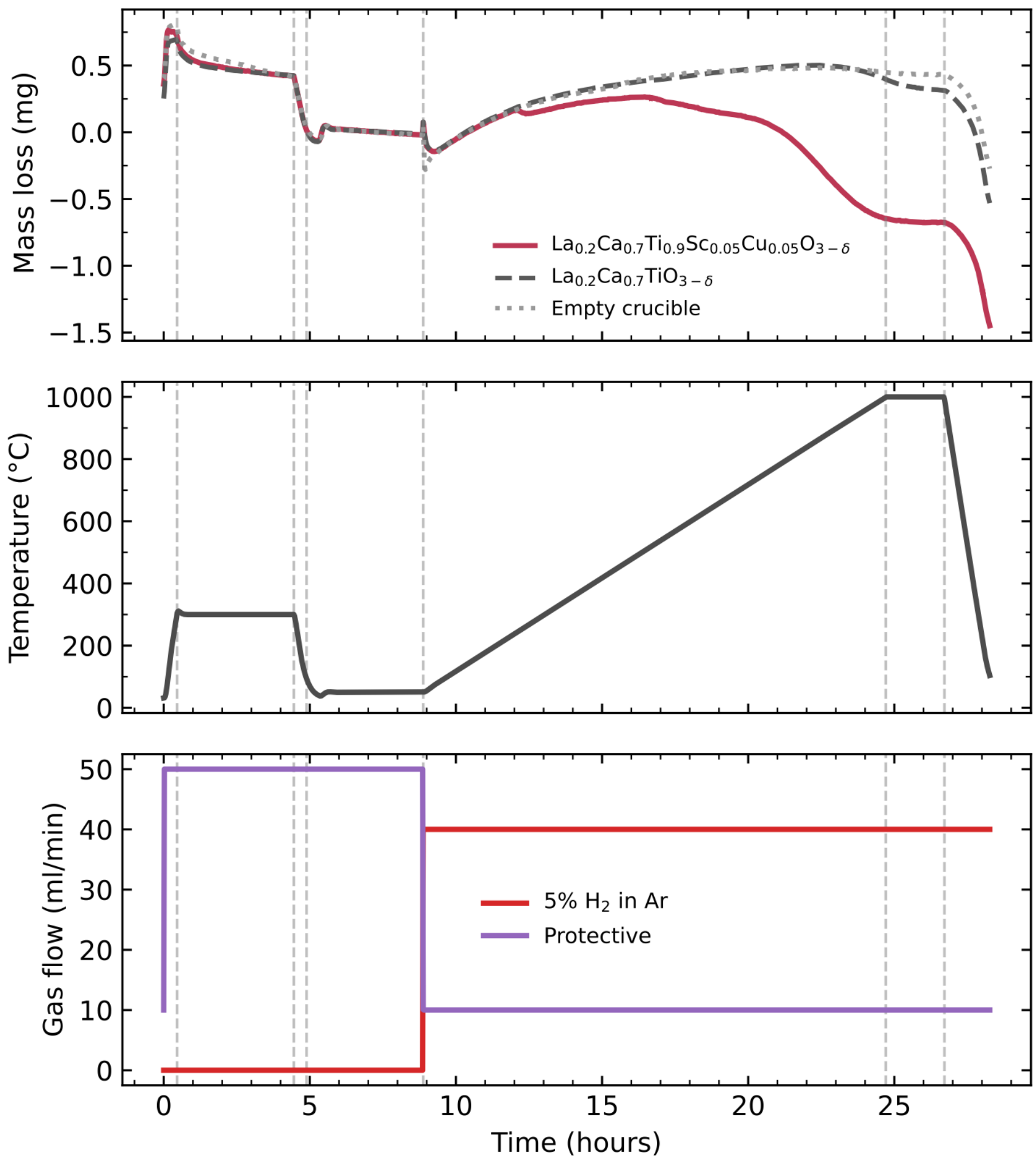

Figure S1. Thermogravimetric analysis of $La_{0.2}Ca_{0.7}Ti_{0.9}Sc_{0.05}Cu_{0.05}O_{3-\delta}$ and $La_{0.2}Ca_{0.7}TiO_{3-\delta}$ reference presented in the manuscript visualized by the measured mass loss (top), temperature profile (middle), and gas flow (bottom). Samples of 20–40 µm sized sieved powders (500 mg) were prepared of $La_{0.2}Ca_{0.7}Ti_{0.9}Sc_{0.05}Cu_{0.05}O_{3-\delta}$ (sintered at 1300 °C) and the $La_{0.2}Ca_{0.7}TiO_{3-\delta}$ reference.

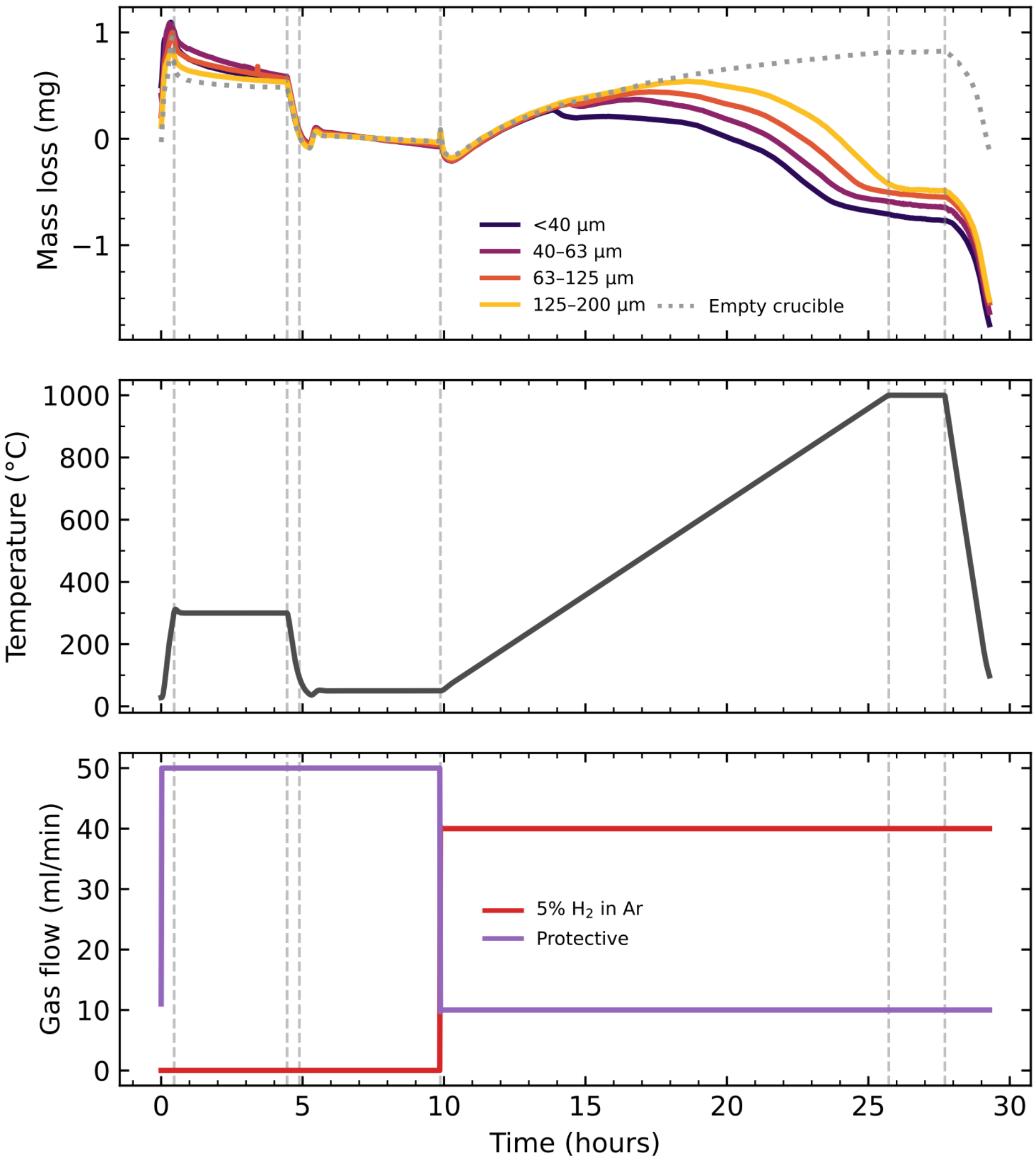

Figure S2. Thermogravimetric analysis of $La_{0.2}Ca_{0.7}Ti_{0.9}Sc_{0.05}Cu_{0.05}O_{3-\delta}$ with increasing particle sizes up to 200 µm diameter visualized by the measured mass loss (top), temperature profile (middle), and gas flow (bottom). Samples of sieved powders with diameters of <40 µm, 40–63 µm, 63–125 µm, and 125–200 µm (500 mg) were prepared of $La_{0.2}Ca_{0.7}Ti_{0.9}Sc_{0.05}Cu_{0.05}O_{3-\delta}$ (sintered at 1500 °C) and the $La_{0.2}Ca_{0.7}TiO_{3-\delta}$ reference (20–40 µm).

2. Structure characterization and refinement (XRD)

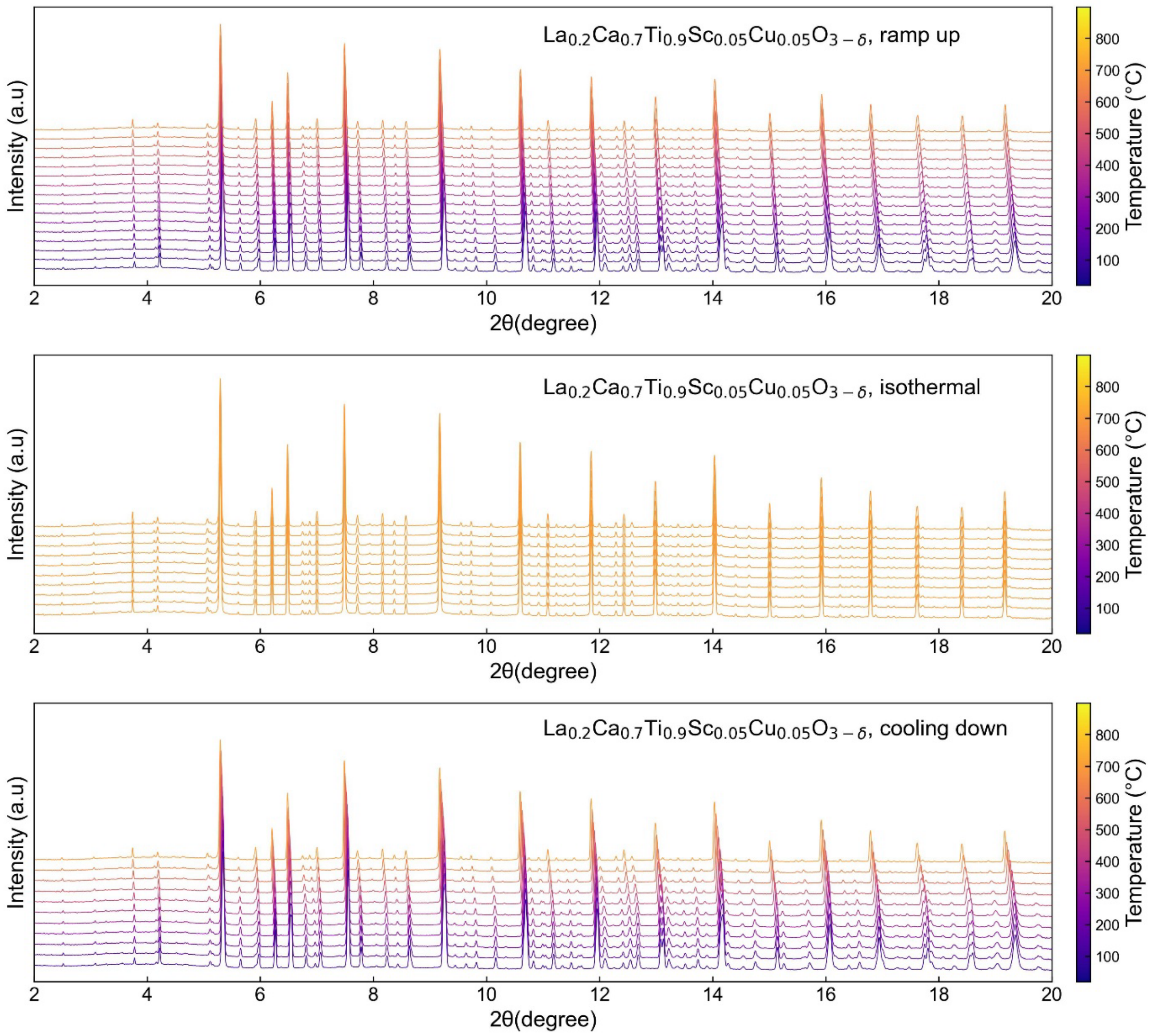

Figure S3. Stacked temperature-dependent XRD patterns on logarithmic scale for $La_{0.2}Ca_{0.7}Ti_{0.9}Sc_{0.05}Cu_{0.05}O_{3-\delta}$.

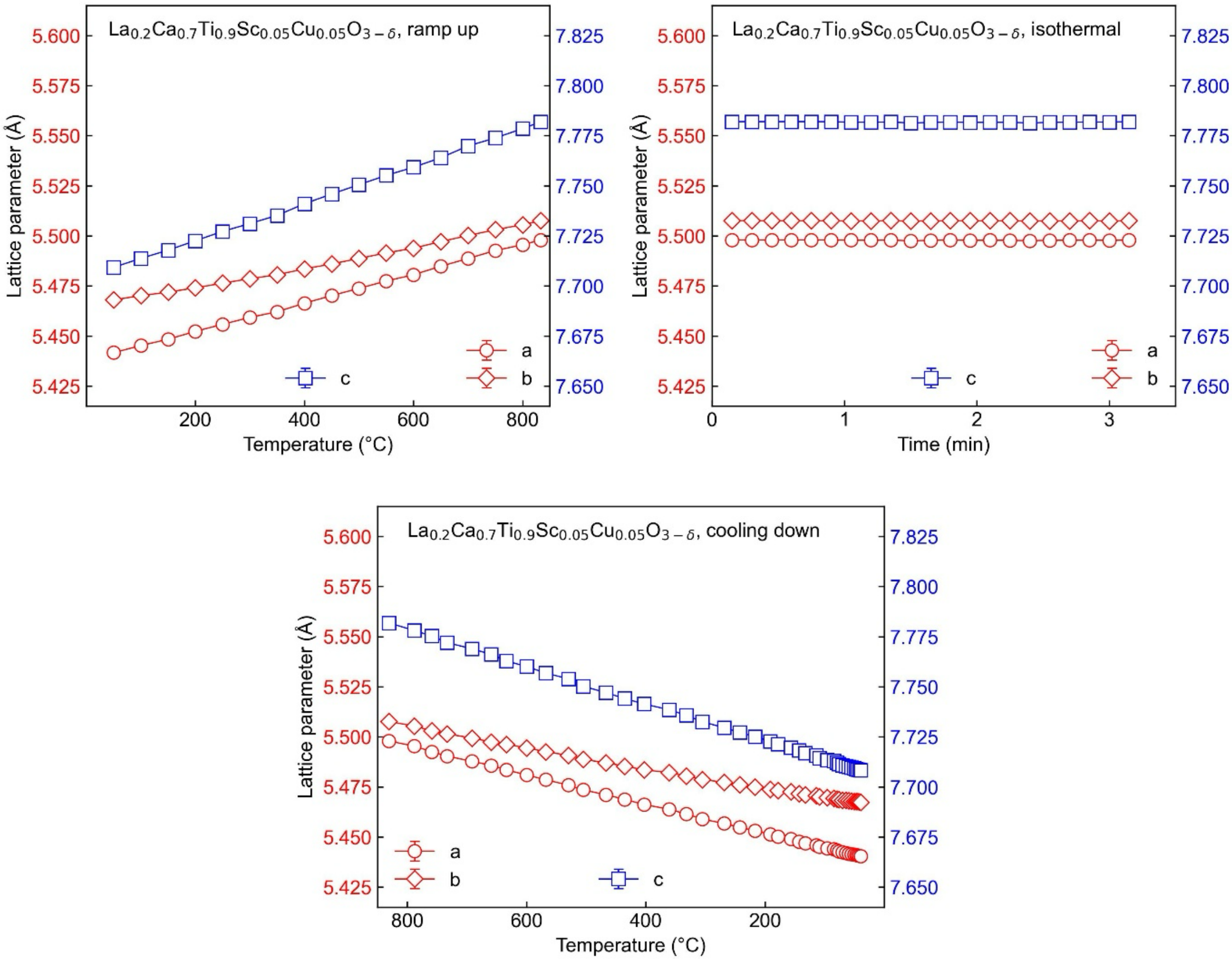

Figure S4. Temperature-dependent evolution of lattice parameters upon heating, isothermal dwell, and cooling for $La_{0.2}Ca_{0.7}Ti_{0.9}Sc_{0.05}Cu_{0.05}O_{3-\delta}$.

Estimation of the metal exsolution yield in percent in **Figure 1c** of the manuscript was performed by the exsolution reaction according to Equation (1):

$$\mathrm{La_{0.2}Ca_{0.7}Ti_{0.9}Sc_{0.05}Cu_{0.05}O_{2.925}} \rightarrow \mathrm{La_{0.2}Ca_{0.7}Ti_{0.9}Sc_{0.05}Cu_{0.05-\mathit{x}}O_{2.925-\mathit{x}}} + x\mathrm{Cu(s)} + \tfrac{x}{2}\mathrm{O_2(g)} \quad (1)$$

The final weight fraction of the exsolved Cu X_f can be expressed after complete exsolution, i.e., $x = 0.05$, as in Equation (2):

$$X_\mathrm{f} = \frac{0.05\ \mathrm{MW_{Cu}}}{0.05\ \mathrm{MW_{Cu}} + \mathrm{MW_{LCT}}} \quad (2)$$

Here, the MW_{LCT} is the molecular weight of the host after exsolution, i.e., without copper and with the corresponding amount of oxygen loss according to Equation 1. The mole fraction of exsolved Cu throughout the in situ experiment was obtained from the weight fraction of Cu obtained from Rietveld refinement (X) relative to the final weight fraction:

$$\frac{x}{0.05} = \frac{X}{X_\mathrm{f}} \quad (3)$$

3. Temperature profiles of in situ microscopy experiments (STEM)

Three in situ STEM experiments were conducted to ensure reproducibility of the findings and evaluate any effects of electron-beam irradiation[1]. Two experiments were conducted on $La_{0.2}Ca_{0.7}Ti_{0.9}Sc_{0.05}Cu_{0.05}O_{3-\delta}$ and one on $La_{0.2}Ca_{0.7}Ti_{0.9}Al_{0.05}Cu_{0.05}O_{3-\delta}$. The employed temperature profiles are presented in **Figure S5**. Chronologically, the experiment on $La_{0.2}Ca_{0.7}Ti_{0.9}Al_{0.05}Cu_{0.05}O_{3-\delta}$ was conducted first, aiming to identify the onset temperatures of the stages under the vacuum conditions (10^{-8} mbar). The wedge was monitored during heating at increments of 50 °C. As a result, the onset of the initial, second, and third stage were identified at 400 °C, 600 °C, and 1000 °C, respectively. Lastly, the upper temperature limit of the microchip was evaluated by heating to 1100 °C to monitor particle migration and coalescence. At this temperature, the carbon film and silicon nitride support started degrading and eventually broke after about an hour. The upper temperature limit for the subsequent experiments was therefore set to 1000 °C, sufficient to activate all stages and for the microchip to remain intact.

The in situ experiment was repeated for $La_{0.2}Ca_{0.7}Ti_{0.9}Sc_{0.05}Cu_{0.05}O_{3-\delta}$ with the aim of correlating with synchrotron and thermogravimetry experiments. The onset temperature of initial and second stages matched that of the previous experiment. The smallest nanoparticles appeared sensitive to the electron beam when monitored at atomic resolution at 450 °C (**Figure S8**g), whereas there were no signs of instability under the electron beam at lower magnification below 600 °C. Further, high magnification imaging was conducted at room temperature after rapid quenching.

Results from the third in situ experiment is presented in the main text, in which measurements were performed at selected temperatures within the range of each stage. The <110>-wedges of $La_{0.2}Ca_{0.7}Ti_{0.9}Sc_{0.05}Cu_{0.05}O_{3-\delta}$ was studied in the third experiment, while the <111>- and <100>-oriented wedges of $La_{0.2}Ca_{0.7}Ti_{0.9}Sc_{0.05}Cu_{0.05}O_{3-\delta}$ and $La_{0.2}Ca_{0.7}Ti_{0.9}Al_{0.05}Cu_{0.05}O_{3-\delta}$ were studied in the preceding experiment, respectively. All images were taken in STEM with either annular bright-field (ABF)[2] or annular dark-field (ADF) imaging with a low inner collection angle to exploit enhanced contrast from lattice imperfections[3,4].

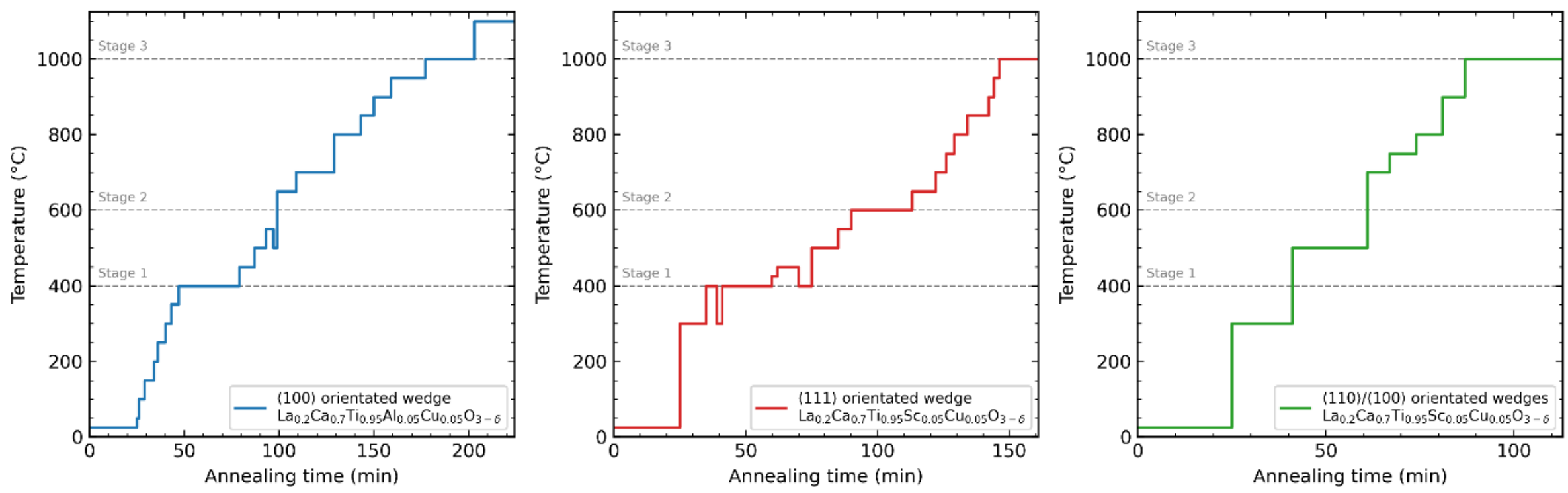

Figure S5. Annealing profiles of the in situ STEM experiments. Annealing temperature and duration for the experiment conducted on $La_{0.2}Ca_{0.7}Ti_{0.9}Al_{0.05}Cu_{0.05}O_{3-\delta}$ (left) and $La_{0.2}Ca_{0.7}Ti_{0.9}Sc_{0.05}Cu_{0.05}O_{3-\delta}$ in the first (middle) and second (right) experiment.

4. Exsolution at the initial stage (STEM)

The pristine wedge together with the first observed and stabilized copper nanoparticles are presented for the wedges in the following order:

- **Figure S6**: <110> oriented wedge of $La_{0.2}Ca_{0.7}Ti_{0.9}Sc_{0.05}Cu_{0.05}O_{3-\delta}$
- **Figure S7**: <100> oriented wedge of $La_{0.2}Ca_{0.7}Ti_{0.9}Al_{0.05}Cu_{0.05}O_{3-\delta}$
- **Figure S8**: <111> oriented wedge of $La_{0.2}Ca_{0.7}Ti_{0.9}Sc_{0.05}Cu_{0.05}O_{3-\delta}$

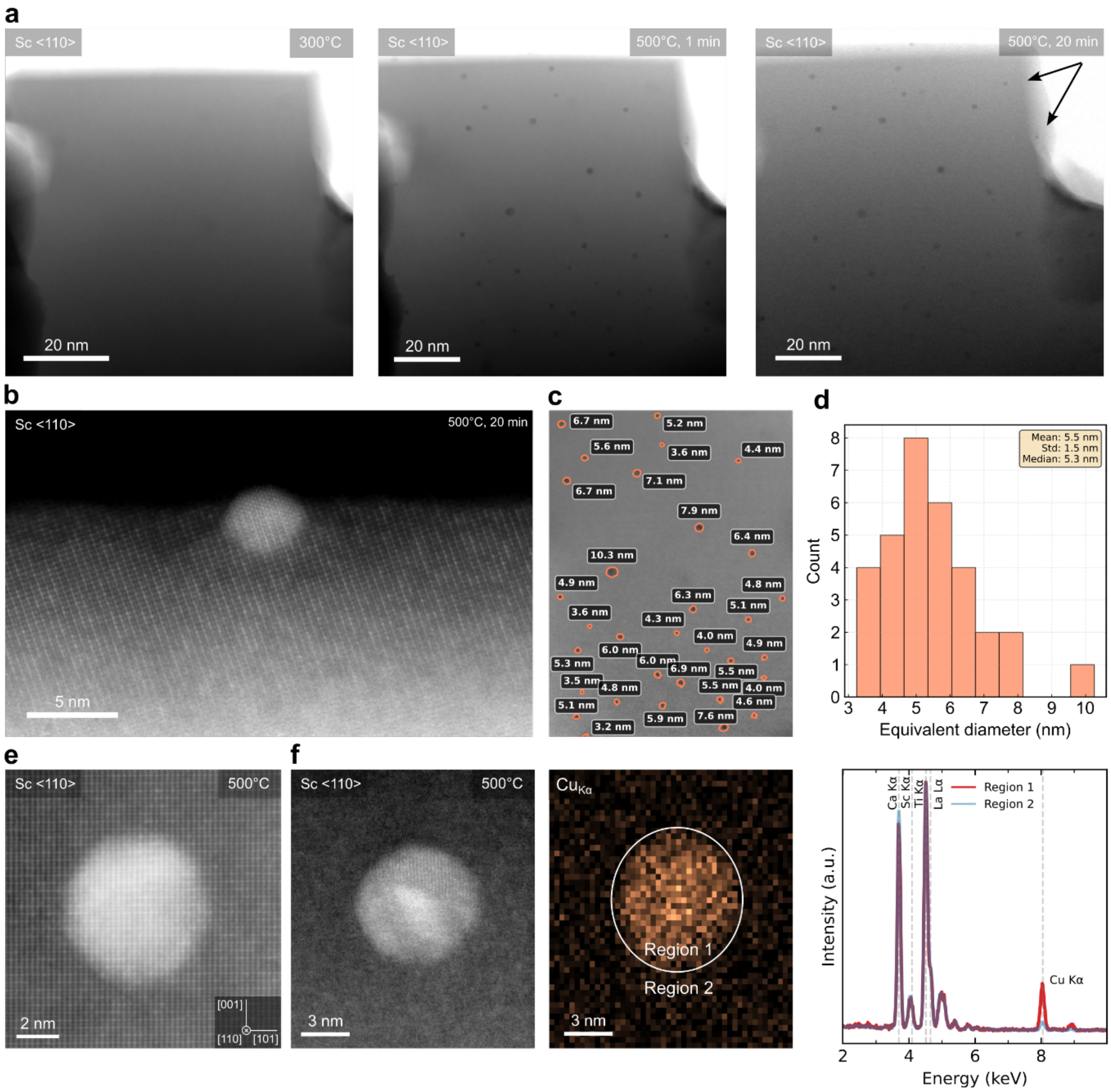

Figure S6. Initial exsolution of <110> oriented wedge of $La_{0.2}Ca_{0.7}Ti_{0.9}Sc_{0.05}Cu_{0.05}O_{3-\delta}$. **a,** Overview STEM ABF image of the wedge at 300 °C (left) and at 500 °C after one and twenty minutes (middle, right). The stabilized particle population show no sign of diffusion. Arrows mark two nucleated particles between the observations. **b,** Atomically resolved STEM ADF image of an anchored surface nanoparticle after twenty minutes at 500 °C. **c-d,** Quantified diameters of the nanoparticle population as marked with annotations in the ABF image and presented as a histogram. **e-f,** STEM ADF and energy dispersive X-ray spectroscopy (EDS) elemental map and overlayed spectra of regions 1 and 2 confirm that the exsolved nanoparticles are metallic copper.

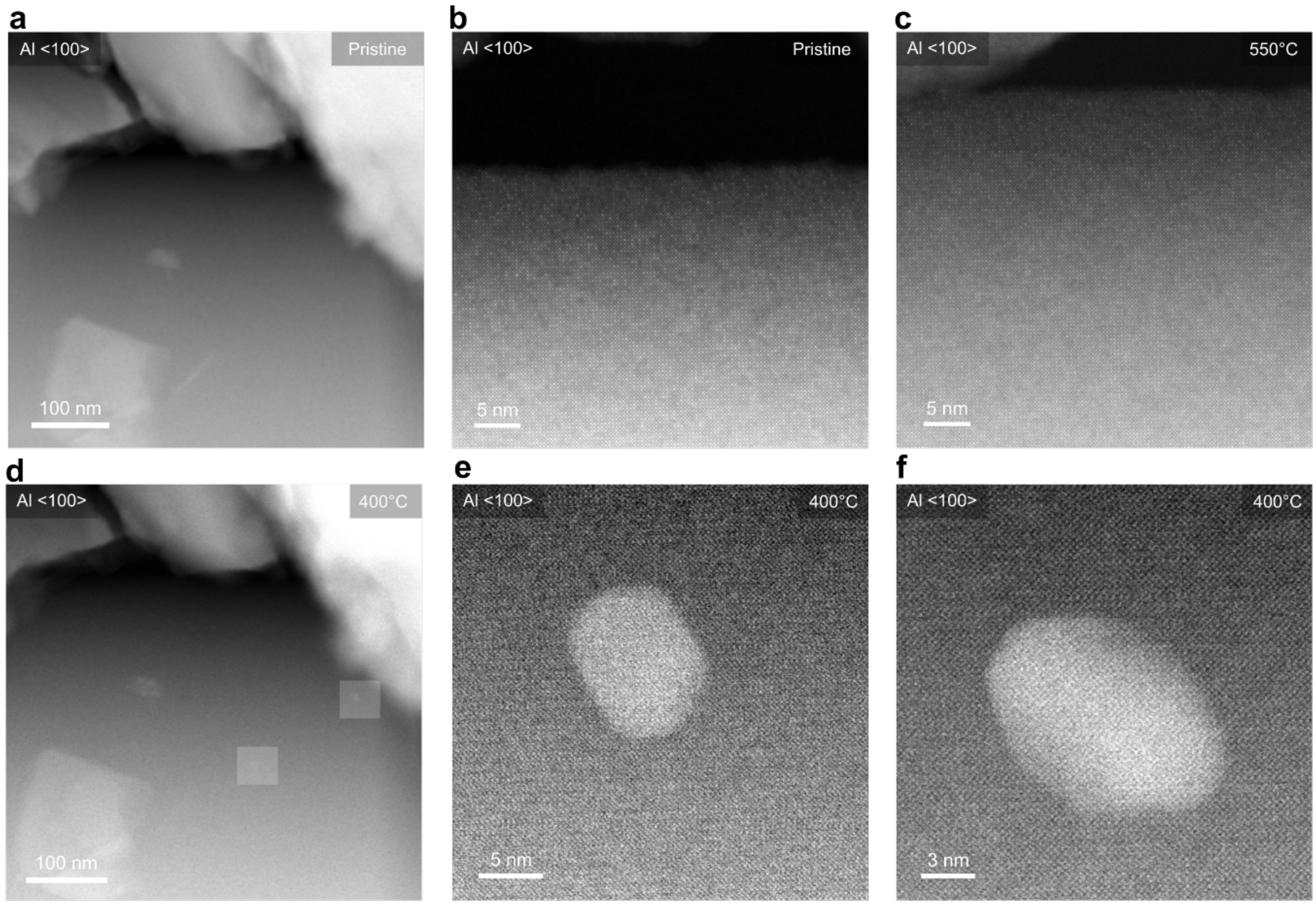

Figure S7. Initial exsolution of <100>-oriented wedges of $La_{0.2}Ca_{0.7}Ti_{0.9}Al_{0.05}Cu_{0.05}O_{3-\delta}$. **a,** Overview STEM ADF image of the pristine wedge. **b-c**, Atomically resolved STEM ADF images of the wedge edge at the pristine state and at 550 °C. **d**, Overview STEM-ADF image of the wedge at 400 °C when the surface particles were first observed. **e-f**, Nanoscale STEM ADF image of nanoparticles of varying shapes.

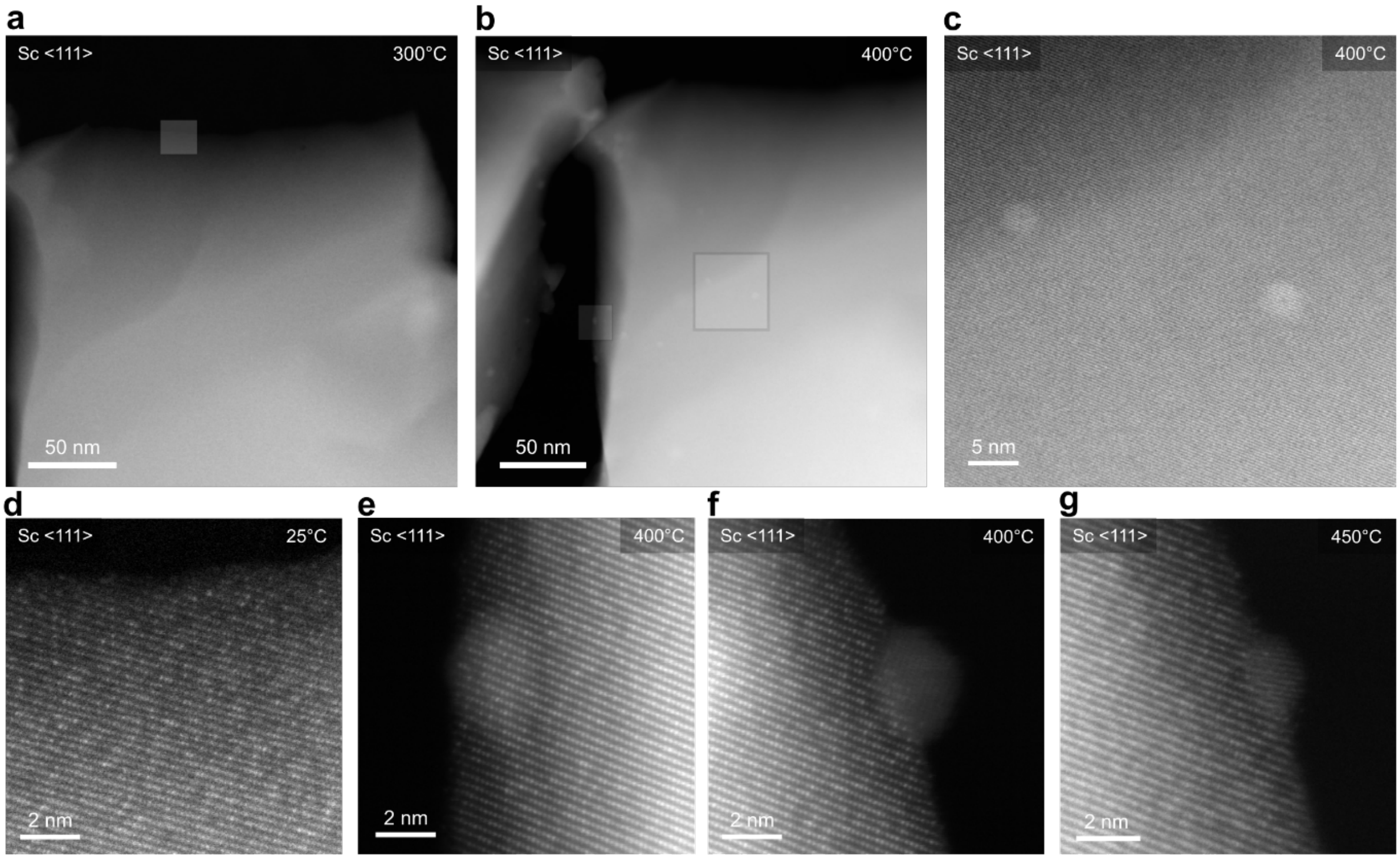

Figure S8. Initial exsolution of <111>-oriented wedges of $La_{0.2}Ca_{0.7}Ti_{0.9}Sc_{0.05}Cu_{0.05}O_{3-\delta}$. **a-b,** Overview STEM ADF images of the wedge at 300 °C and 400 °C when particles are first detected. **c,** Nanoscale image of the nanoparticles as marked by the larger square in b. **d,** Atomically resolved STEM-ADF image of the pristine wedge. **e-g,** Atomically resolved STEM-ADF images of surface nanoparticles at 400–450 °C.

5. Endoparticle formation and surface reconstruction (STEM)

Reconstruction of the perovskite surface by formation of steps and terraces was also observed during the second experiment on the <111>- oriented wedges of $La_{0.2}Ca_{0.7}Ti_{0.9}Sc_{0.05}Cu_{0.05}O_{3-\delta}$. The evolution of the perovskite surface and endoparticle formation is summarized in **Figure S9**.

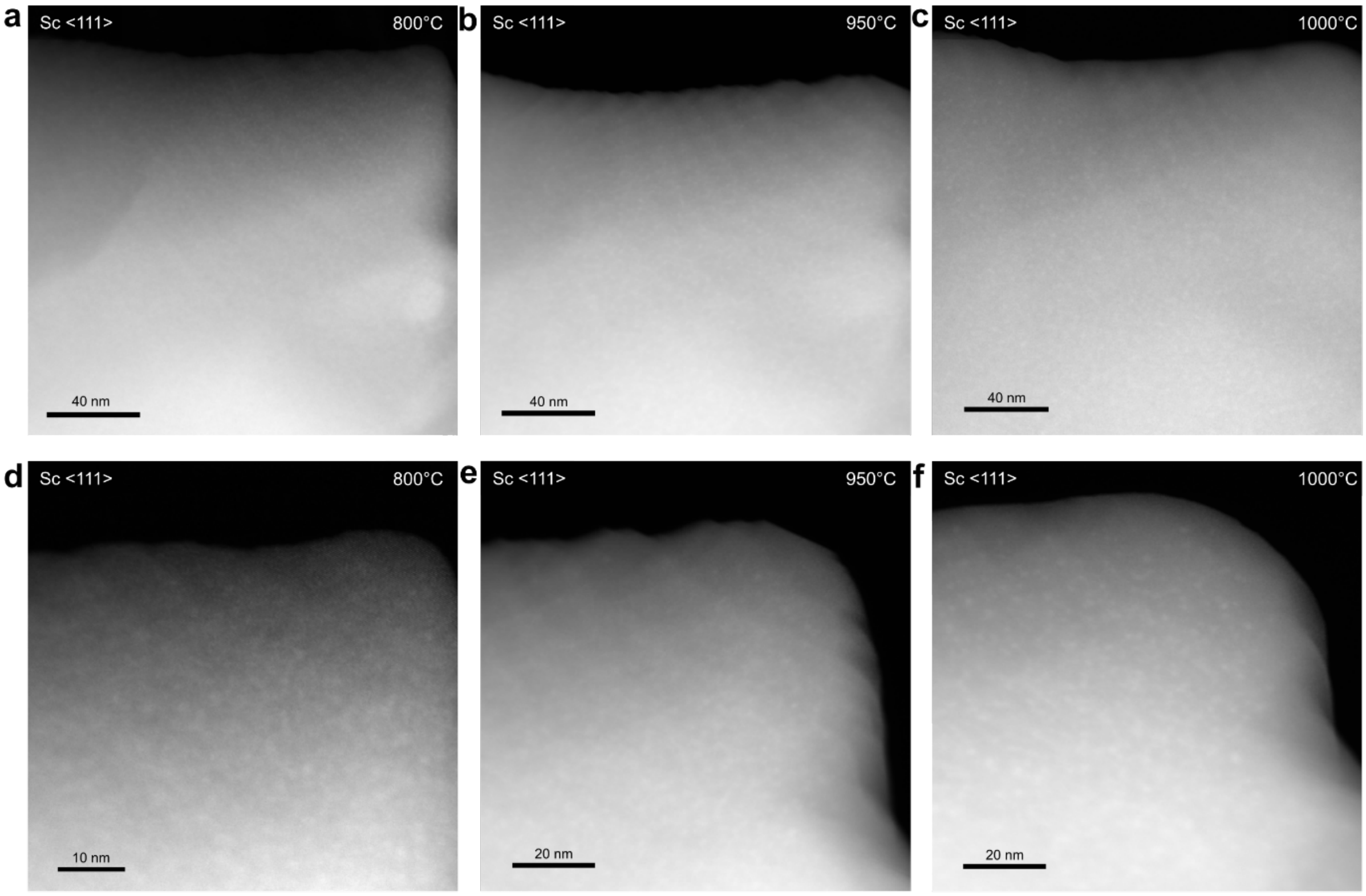

Figure S9. Reproduced surface reconstruction with endoparticle growth on <111>- oriented wedge of $La_{0.2}Ca_{0.7}Ti_{0.9}Sc_{0.05}Cu_{0.05}O_{3-\delta}$. **a-c**, Overview STEM-ADF image of the wedge at 800 °C, 950 °C, and 1000 °C show large scale surface transformation concurrent with endoparticle exsolution. **d-f**, STEM-ADF image of the wedge edge show periodic contrast in the perovskite at 800 °C, prior to the spontaneous surface reconstruction at 950 °C and later surface smoothening at 1000 °C.

6. Diffusion and coalescing in the bulk and at the surface (STEM)

The in situ STEM observations for the nanoparticle population observed at 1000 °C when the perovskite surface is smoothed were reproduced for $La_{0.2}Ca_{0.7}Ti_{0.9}Al_{0.05}Cu_{0.05}O_{3-\delta}$ (**Figure S10**). Elemental maps representing peak intensity from the model fitting within eXSpy ecosystem of the hyperspy package[5] confirm the ADF contrast correlate to the $Cu_{K\alpha}$ signal (**Figure S11)**. The elemental maps from the other cations using $Ca_{K\alpha}$, $La_{L\alpha}$, $Ti_{K\alpha}$, and $Sc_{K\alpha}$ show intensity distributions correlated to the thickness of the wedge with minor spatial inhomogeneity that are not correlated to the STEM ADF contrast due to the relatively limited size of the dataset. Migration and coalescing of nanoparticles was observed at 1100 °C at the <100>- oriented wedges of $La_{0.2}Ca_{0.7}Ti_{0.9}Al_{0.05}Cu_{0.05}O_{3-\delta}$ (**Figure S12**).

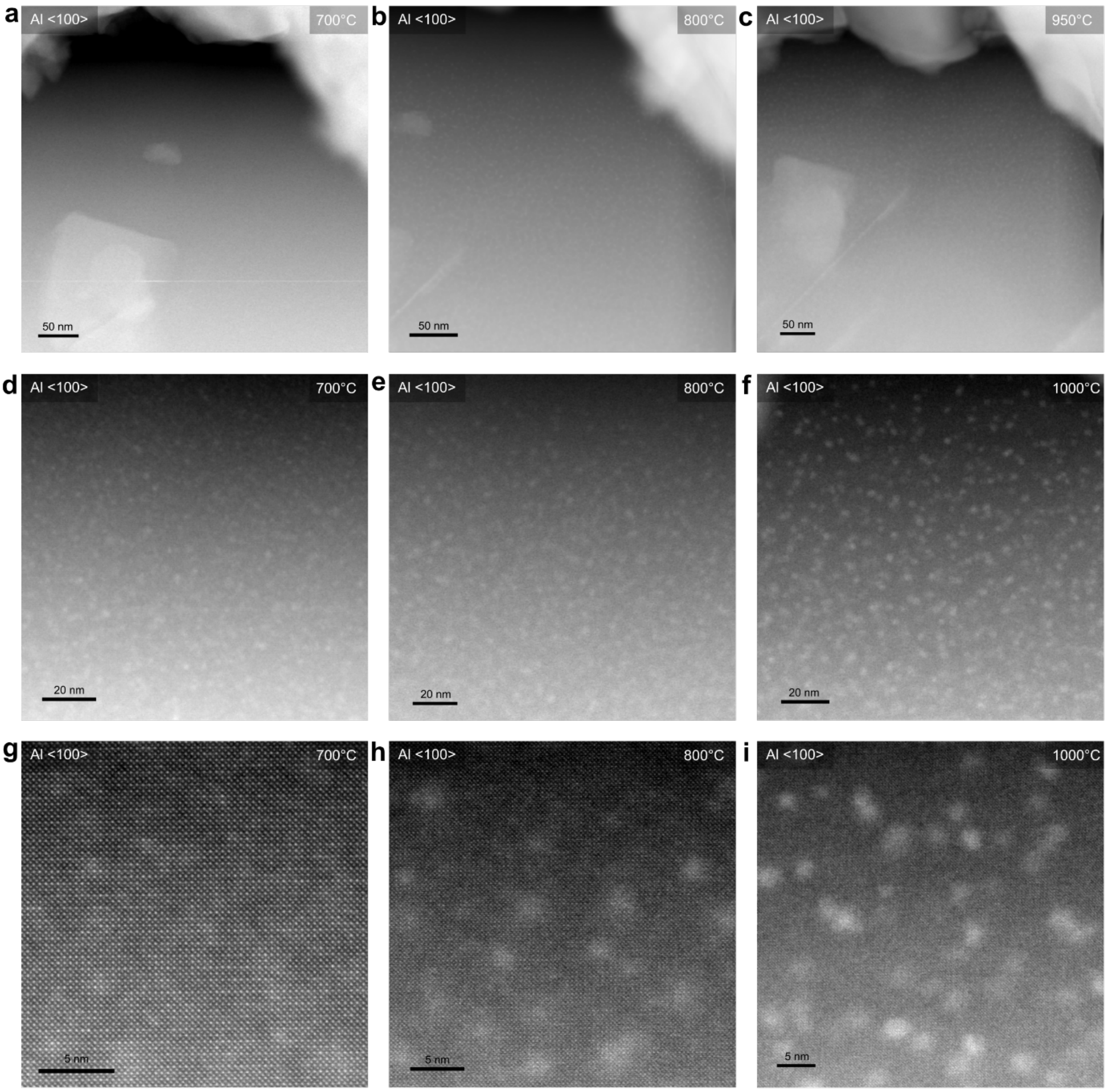

Figure S10. Exsolution above 600 °C of <100>- oriented wedges of $La_{0.2}Ca_{0.7}Ti_{0.9}Al_{0.05}Cu_{0.05}O_{3-\delta}$. **a-c,** Low- and **d-f**, high-magnification as well as **g-i**, atomically resolved STEM-ADF image of the wedge at 700 °C, 800 °C, and 950 °C show endoparticle formation and growth. The developing line of contrast in **a–c** is an initial crack in the wedge which becomes filled by copper particles, and moves into the image as the particle wedge tilts during the experiment.

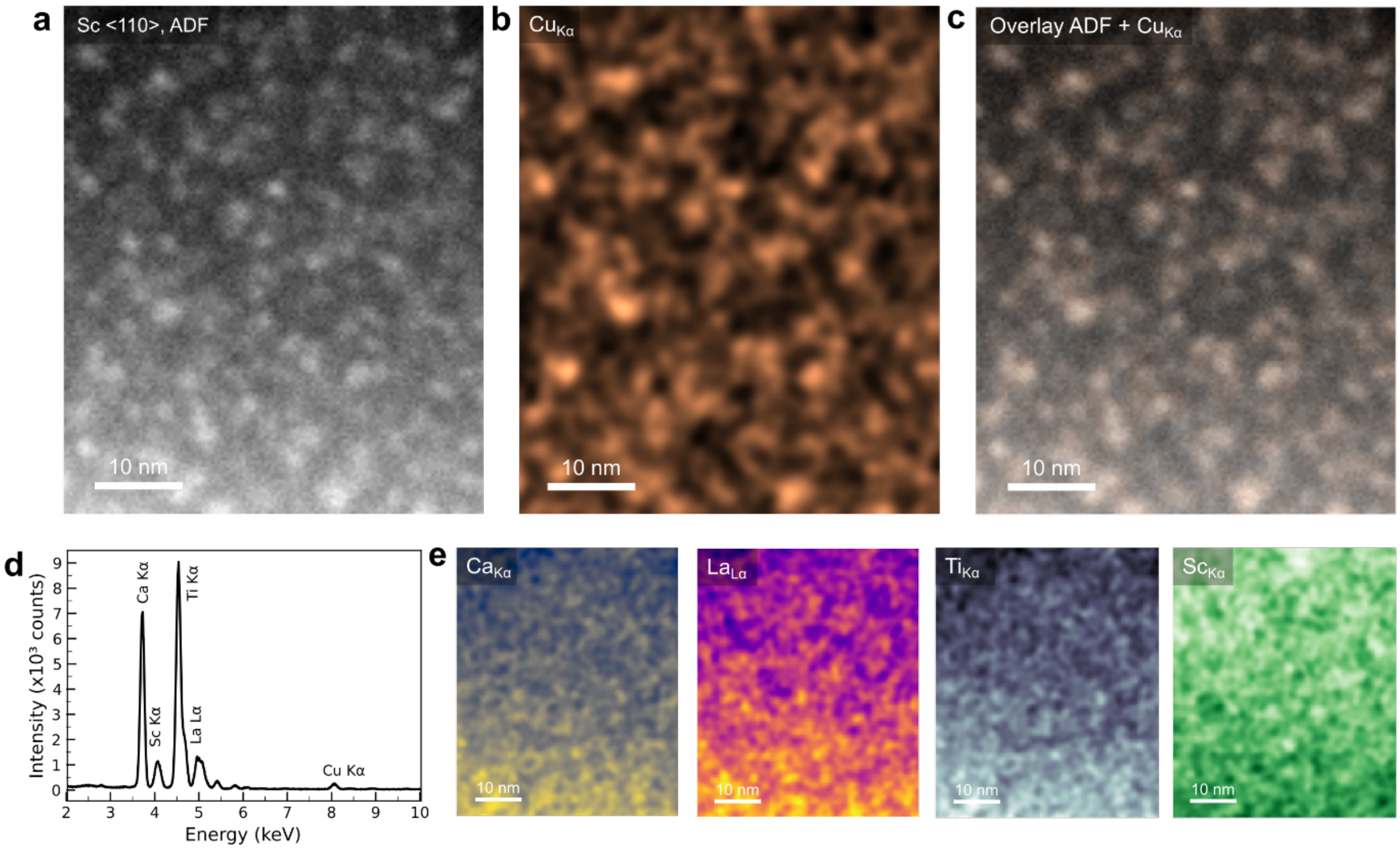

Figure S11. STEM energy dispersive spectroscopy analysis of copper population of <110> oriented wedge of $La_{0.2}Ca_{0.7}Ti_{0.9}Sc_{0.05}Cu_{0.05}O_{3-\delta}$ after annealed at 1000 °C. **a,** STEM annular dark field (ADF) image of the exsolved particle population with **b,** elemental map of $Cu_{K\alpha}$ fitting results in the same region. **c,** Strong correlation in the overlayed STEM-ADF image and $Cu_{K\alpha}$ elemental map confirms that the image contrast originates from copper. **d,** Summed EDS spectra show the cation X-ray lines of interest. **e,** Elemental map of $Ca_{K\alpha}$, $La_{L\alpha}$, $Ti_{K\alpha}$, and $Sc_{K\alpha}$. Preprocessing was performed using principal component analysis[6] to remove noise. Gaussian kernel to smooth the signal in nearby pixels was conducted prior to the model fitting to improve signal to noise and background.

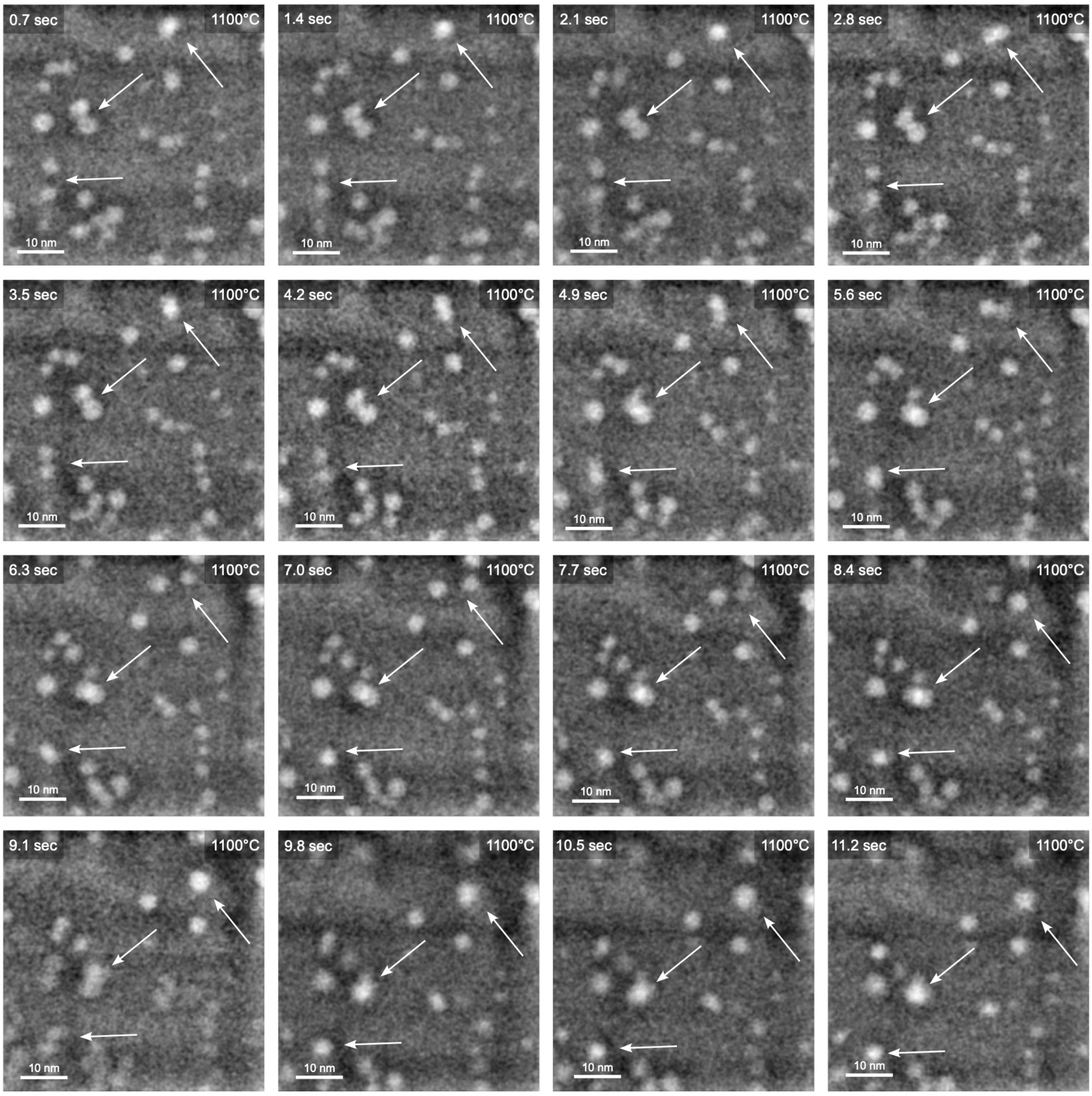

Figure S12. Migration and coalescence of surface nanoparticles at 1100 °C of <100>-oriented wedges of $La_{0.2}Ca_{0.7}Ti_{0.9}Al_{0.05}Cu_{0.05}O_{3-\delta}$. 16 second STEM-ADF image series show nanoparticle migration and coalescence as the three examples marked with the arrows. The image series is post-processed using adaptive histogram equivalence to enhance contrast, a low pass Fourier filter to remove noise, and a Gaussian filter to smooth the image by the use of the SciPy package[7].

References

1. Sarkar, R., Rentenberger, C. & Rajagopalan, J. Electron Beam Induced Artifacts During in situ TEM Deformation of Nanostructured Metals. *Sci Rep* **5**, 16345 (2015).
2. Findlay, S. D. *et al.* Dynamics of annular bright field imaging in scanning transmission electron microscopy. *Ultramicroscopy* **110**, 903–923 (2010).
3. Phillips, P. J. *et al.* Atomic-resolution defect contrast in low angle annular dark-field STEM. *Ultramicroscopy* **116**, 47–55 (2012).
4. Muller, D. A., Nakagawa, N., Ohtomo, A., Grazul, J. L. & Hwang, H. Y. Atomic-scale imaging of nanoengineered oxygen vacancy profiles in SrTiO3. *Nature* **430**, 657–661 (2004).
5. Peña, F. de la *et al.* hyperspy/hyperspy: v2.2.0. Zenodo https://doi.org/10.5281/zenodo.14057415 (2024).
6. Greenacre, M. *et al.* Principal component analysis. *Nat Rev Methods Primers* **2**, 100 (2022).
7. Virtanen, P. *et al.* SciPy 1.0: fundamental algorithms for scientific computing in Python. *Nat Methods* **17**, 261–272 (2020).
